# Comparative transcriptome analysis of oil palm flowers reveals an EAR-motif-containing R2R3-MYB that modulates phenylpropene biosynthesis

**DOI:** 10.1186/s12870-017-1174-4

**Published:** 2017-11-23

**Authors:** Ran Li, Vaishnavi Amarr Reddy, Jingjing Jin, Chakaravarthy Rajan, Qian Wang, Genhua Yue, Chin Huat Lim, Nam-Hai Chua, Jian Ye, Rajani Sarojam

**Affiliations:** 1 Temasek Life Sciences Laboratory, National University of Singapore, Singapore, 117604 Singapore; 2 Department of Biological Sciences, National University of Singapore, Singapore, 117543 Singapore; 3 R&D Department, Wilmar International Plantation, Palembang, Indonesia; 4 Laboratory of Plant Molecular Biology, Rockefeller University, New York, NY 10065 USA; 5 State Key Laboratory of Plant Genomics, Institute of Microbiology, Chinese Academy of Sciences, Beijing, 100101 China; 6 Present Address: Department of Molecular Ecology, Max Planck Institute for Chemical Ecology, 07745 Jena, Germany; 7 Present Address: Singapore Centre on Environmental Life Sciences Engineering, Nanyang Technological University, Singapore, Singapore; 8 Present Address: College of Biological and Environmental Sciences, Zhejiang Wanli University, Ningbo, Zhejiang, China

**Keywords:** MYB transcription factor, Phenylpropene, Lignin, Oil palm, Basil, Phenylpropanoid pathway

## Abstract

**Background:**

Oil palm is the most productive oil crop and the efficiency of pollination has a direct impact on the yield of oil. Pollination by wind can occur but maximal pollination is mediated by the weevil *E. kamerunicus.* These weevils complete their life cycle by feeding on male flowers. Attraction of weevils to oil palm flowers is due to the emission of methylchavicol by both male and female flowers. In search for male flowers, the weevils visit female flowers by accident due to methylchavicol fragrance and deposit pollen. Given the importance of methylchavicol emission on pollination, we performed comparative transcriptome analysis of oil palm flowers and leaves to identify candidate genes involved in methylchavicol production in flowers.

**Results:**

RNA sequencing (RNA-Seq) of male open flowers, female open flowers and leaves was performed using Illumina HiSeq 2000 platform. Analysis of the transcriptome data revealed that the transcripts of methylchavicol biosynthesis genes were strongly up-regulated whereas transcripts encoding genes involved in lignin production such as, *caffeic acid O*-*methyltransferase* (*COMT*) and *Ferulate*-*5*-*hydroxylase* (*F5H*) were found to be suppressed in oil palm flowers. Among the transcripts encoding transcription factors, an EAR-motif-containing R2R3-MYB transcription factor (*EgMYB4*) was found to be enriched in oil palm flowers. We determined that EgMYB4 can suppress the expression of a monolignol pathway gene, *EgCOMT*, in vivo by binding to the AC elements present in the promoter region. *EgMYB4* was further functionally characterized in sweet basil which also produces phenylpropenes like oil palm. Transgenic sweet basil plants showed significant reduction in lignin content but produced more phenylpropenes.

**Conclusions:**

Our results suggest that *EgMYB4* possibly restrains lignin biosynthesis in oil palm flowers thus allowing enhanced carbon flux into the phenylpropene pathway. This study augments our understanding of the diverse roles that EAR-motif-containing MYBs play to fine tune the metabolic flux along the various branches of core phenylpropanoid pathway. This will aid in metabolic engineering of plant aromatic compounds.

**Electronic supplementary material:**

The online version of this article (10.1186/s12870-017-1174-4) contains supplementary material, which is available to authorized users.

## Background

Oil palm (*Elaeis guineensis*) is one of the most important monocot cash crop of Southeast Asia [1, 2]. Palm oil derived from its fruits is the largest source of edible vegetable oil in the world [3]. It was first introduced in Southeast Asia in 1848 and was planted on a commercial scale around 1917. The subsequent accelerated planting of palm trees and expansion of oil palm plantations boosted the economies of the developing Southeast Asian countries [4]. Oil palm is monoecious, producing male and female flowers on the same tree but at different times. To produce fruits, the plant needs to attract efficient pollinators to its flowers. *Elaeidobius kamerunicus*, a type of weevil which originated from West Africa, is considered the most competent and dominant insect pollinator species of oil palm. Introduction of this weevil into Malaysia during the 1980s enhanced the pollination rate of oil palm trees leading to 20–30% increase in fruit production [5]. To attract the weevil for pollination, both male and female flowers release a volatile phenylpropene compound called methylchavicol (also known as estragole) [6].

**Fig. 1:**
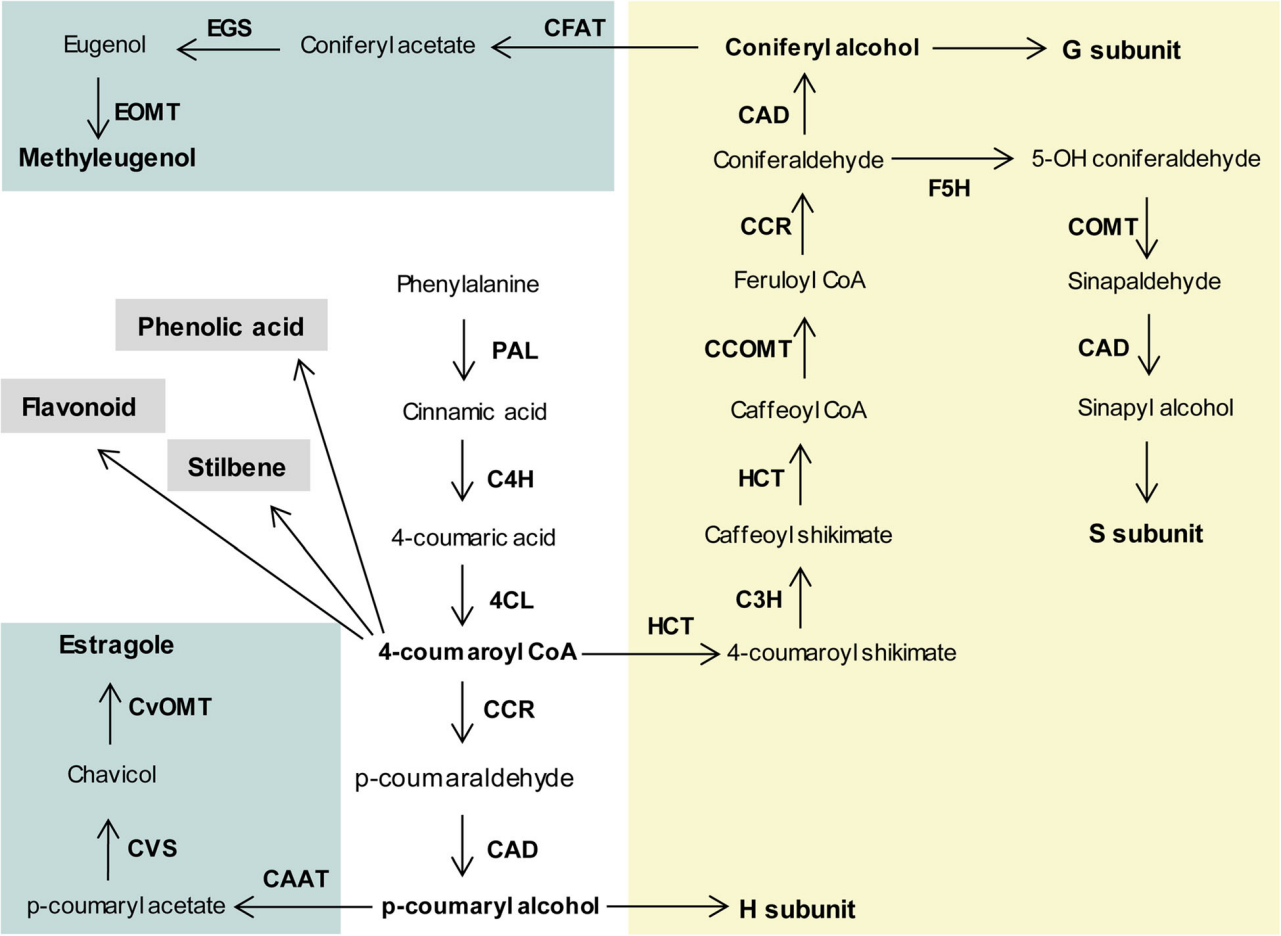
The phenylpropanoid pathway in plant. CAAT, coumaryl alcohol acetyl transferase; CAD, cinnamyl alcohol dehydrogenase; CFAT, coniferyl alcohol acetyl transferase; CCOMT, caffeoyl-CoA O-methyltransferase; CCR, cinnamoyl-CoA reductase; C3H, p-coumaroylshikimate 3′-hydroxylase; C4H, cinnamate 4-hydroxylase; 4CL, 4-coumaroyl CoAligase; COMT, caffeic acid O-methyltransferase; CvOMT, chavicol O-methyltransferase; CVS, chavicol synthase; EGS, eugenol synthase; EOMT, eugenol O-methyltransferase; F5H, ferulate-5-hydroxylase; HCT, hydroxycinnamoyl-CoA shikimate/quinate hydroxycinnamoyl transferase; PAL, phenylalanine ammonia lyase

Phenylpropenes (C6-C3 carbon skeleton) are a class of volatile organic compounds (VOCs) produced by plants that serve as pollinator attractors and aid in pathogen defense [7, 8]. Commonly produced phenylpropenes include chavicol and eugenol, and their derivatives methylchavicol, methyleugenol and isoeugenol. Phenylpropenes are produced by the general phenylpropanoid pathway in plants [9]. This pathway is also responsible for the production of lignins, flavonoids, phenolic acids and stilbenes ([10]; Fig. 1). Among these phenylpropanoid derived metabolites, lignin is vital for plant growth and development. It enhances cell wall strength and is required for water transport and mechanical strength [11]. Lignin is a polymer derived from three monolignols, p-coumaryl, coniferyl and sinapyl alcohol. These three precursors produce the hydroxyphenyl (H), guaiacyl (G) and syringyl (S) units of lignin respectively (Fig. 1; [12]). Phenylpropenes are also produced from monolignols; for instance, chavicol is made from p-coumaryl alcohol, whereas, eugenol is derived from coniferyl alcohol (Fig. 1; [13]). Hence, plant tissues producing phenylpropenes, such as flowers, need to prudently regulate carbon flux more into scent production than towards lignin formation.

Lignin biosynthesis is extensively controlled by R2R3-MYB transcription factors. AC elements, which serve as the binding sites for R2R3-MYBs, have been found to be enriched in the promoter regions of many lignin biosynthesis genes [14–16]. Several R2R3-MYBs function as activators of lignin synthesis. However, members of the R2R3-MYB subgroup 4 that contain an EAR motif act as transcriptional repressors and some have been identified as negative regulators of lignin production [10, 17, 18]. Perturbations in the expression levels of many of these repressors not only affected the final lignin content but also changed the flux along the various phenylpropanoid pathways. The first R2R3-MYBs characterized to down regulate lignification were *AmMYB308* and *AmMYB330* from *Antirrhinum majus*. Overexpression of *AmMYB308* or *AmMYB330* in tobacco repressed both lignin and phenolic acid metabolism [19]. From Arabidopsis, *AtMYB4* and *AtMYB32* were identified as repressors of lignin biosynthesis genes [20, 21]. The main target of AtMYB4 was shown to be *cinnamate 4-hydroxylase* (*C4H*), a core enzyme of the phenylpropanoid pathway, and AtMYB4 mutants showed enhanced accumulation of sinapate esters. *AtMYB32* has been suggested to be a repressor of Arabidopsis *caffeic acid O*-*methyltransferase* (*COMT*) gene. *Eucalyptus gunnii EgMYB1* affected lignin formation and repressed the expression of two key lignin genes *EgCCR* (cinnamoyl-CoA reductase) and *EgCAD2* (cinnamyl alcohol dehydrogenase) [22]. Further, heterologous expression of chrysanthemum *CmMYB1*, reduced lignin and flavonoid production in Arabidopsis [23].

From monocots, a few subgroup 4 R2R3-MYBs have been characterized as repressors of lignin genes. Maize *ZmMYB31* and *ZmMYB42* downregulate both maize and Arabidopsis *COMT* genes [24]. Ectopic expression of maize *ZmMYB42* in Arabidopsis decreased lignin content and suppressed flavonoid production as well [25]. But ectopic expression of maize *ZmMYB31* in Arabidopsis led to decreased lignin content and increased anthocyanin levels due to the redirection of carbon flux towards anthocyanin pathway. Apart from the *COMT* promoter, it was also shown that ZmMYB31 could bind to the promoter of maize *Ferulate*-*5*-*hydroxylase* (*F5H*) gene in vivo [26]. Recently it was found that *COMT* is a common target of MYB31 and MYB42 in the mature leaves of maize, sorghum and rice. MYB31 and MYB42 were shown to target other genes involved in lignin pathway as well but in a more species-specific manner [27]. *ZmMYB11* from maize was identified based on the sequence similarity with *ZmMYB31* and *ZmMYB42,* and shown to regulate lignin metabolism by binding to *COMT* promoter [18]. A switchgrass (*Panicum virgatum*) ortholog of *AtMYB4, PvMYB4* was shown to negatively regulate lignin formation in both tobacco and switchgrass [16]. Recently from banana, *MusaMYB31* was identified as a repressor of lignin and polyphenols. It was able to down regulate many genes involved in lignin and general phenylpropanoid pathway [28].

In comparison to lignin biosynthesis, very little is known about the regulation of volatile phenylpropenes in plants. Only few TFs have been reported to regulate the production of phenylpropenes. In *Petunia*, R2R3-MYBs, *ODORANT1* (*ODO1*), *EMISSION OF BENZENOIDS II* (*EOBII*) and *PhMYB4*, were identified as regulators of volatile benzenoid/phenylpropanoid compounds in petals [29–33]. Both *PhODO1* and *PhEOBII* function as positive regulators of various shikimate and phenylpropanoid pathway genes while *PhMYB4* acts as a repressor of *C4H* gene and indirectly affects the formation of petunia floral volatiles. *Production of Anthocyanin Pigment1* (*PAP1*) *MYB* transcription factor from Arabidopsis when ectopically expressed in *Petunia* or rose increased the production of phenylpropenes [34, 35]. Recently, *FaEOBII* was identified to control the production of eugenol in ripe strawberry fruits. In *FaEOBII-*silenced plants, the expression of *CAD* and *Eugenol synthase 2 (FaEGS2)* was down-regulated [36]. However, regulation of biosynthesis of volatile phenylpropenes in monocot plants have not been investigated.

In this study, we performed transcriptome sequencing of oil palm leaves, male flowers and female flowers and did a comparative study to identify the genes involved in methylchavicol production. From the differentially expressed TFs, we identified an EAR-motif-containing R2R3-MYB gene, *EgMYB4* (*Elaeis guineensis MYB4*), which was highly enriched in open flowers. Expression of *EgMYB4* coincided with the spatial and developmental production of methylchavicol. Promoter studies showed that *EgMYB4* was able to bind to the promoter of oil palm *EgCOMT* (*caffeic acid O-methyltransferase*) and suppress its expression. To further characterize the function of *EgMYB4*, it was ectopically expressed in sweet basil (*Ocimum basilicum*) plants which produce high quantities of phenylpropenes similar to oil palm. Overexpression of *EgMYB4* decreased lignin content and enhanced the production of phenylpropenes in transgenic sweet basil plants. This suggests that, *EgMYB4* presumably suppresses lignin production in oil palm flowers and redirects the carbon flux to phenylpropene production to promote successful pollination.

## Results

### Methylchavicol is produced at open flower stage of oil palm

**Fig. 2:**
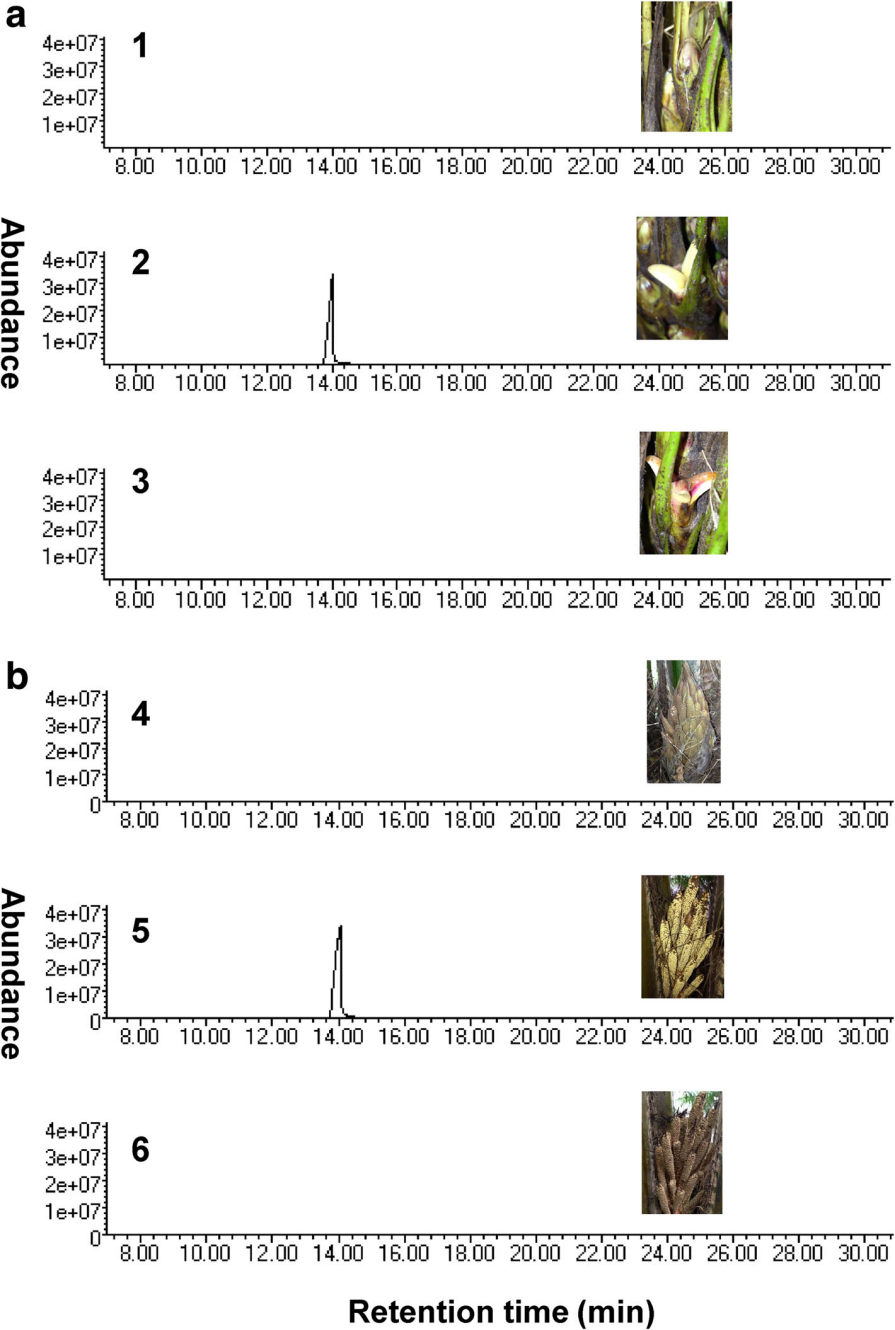
Analysis of methylchavicol in different flower stages. **a** GC-MS analysis of methylchavicol emission in female flower. 1, before anthesis; 2, open flower; 3, after anthesis. **b** GC-MS analysis of methylchavicol emission in male flower. 4, before anthesis; 5, open flower; 6, after anthesis

In oil palm plantations, open flowers give a strong odor like aniseed which is attributed to the emission of methylchavicol [37, 38]. Analysis of male flower, female flower and leaf samples by GC-MS confirmed that the strong odor from open flowers was due to methylchavicol emission (Additional file 1). In leaves, only one green leaf volatile, 2-hexenal, was detected. To investigate methylchavicol emission during different stages of flower development, volatile compounds from three developmental stages of both male and female flowers were extracted and analyzed by GC-MS. The developmental stages analyzed were; before anthesis stage (closed bud), at anthesis stage (newly opened) and after anthesis stage (old flowers). Our results showed that methylchavicol is strongly emitted in male and female flowers at the open flower stage (Fig. 2a and b).

### Sequencing, de novo assembly and annotation of transcriptome

To elucidate methylchavicol biosynthesis in oil palm, we performed RNA-sequencing (RNA-Seq) of male open flowers, female open flowers and leaves using Illumina HiSeq 2000. More than 22 million high quality reads of 101 base pairs from different tissues were generated. The quality of Illumina sequencing outputs was high, as evaluated by FastQC (http://www.bioinformatics.babraham.ac.uk/projects/fastqc/) (Additional file 2). Using the Trinity method (default parameter) [39], the sequence reads were finally assembled into 59,078 non-redundant unigenes with 96,062 isoforms (with N50 = 1884 bp and mean size = 1104 bp). All unigenes were longer than 200 bp. The unigenes were annotated by performing a BLASTX search against various protein databases including TAIR and UniProt (e-value bigger than 1e-3). Functions were assigned by top hit candidates. Expression levels for assembled transcripts were calculated by mapping the reads onto them using Bowtie [40]. Later, by using RSEM (RNA-Seq by Expectation-Maximization), the abundance of assembled transcripts was estimated and the expression levels were measured [41].

### EgMYB4 is an EAR-motif-containing R2R3-MYB TF that is enriched in flowers

**Fig. 3:**
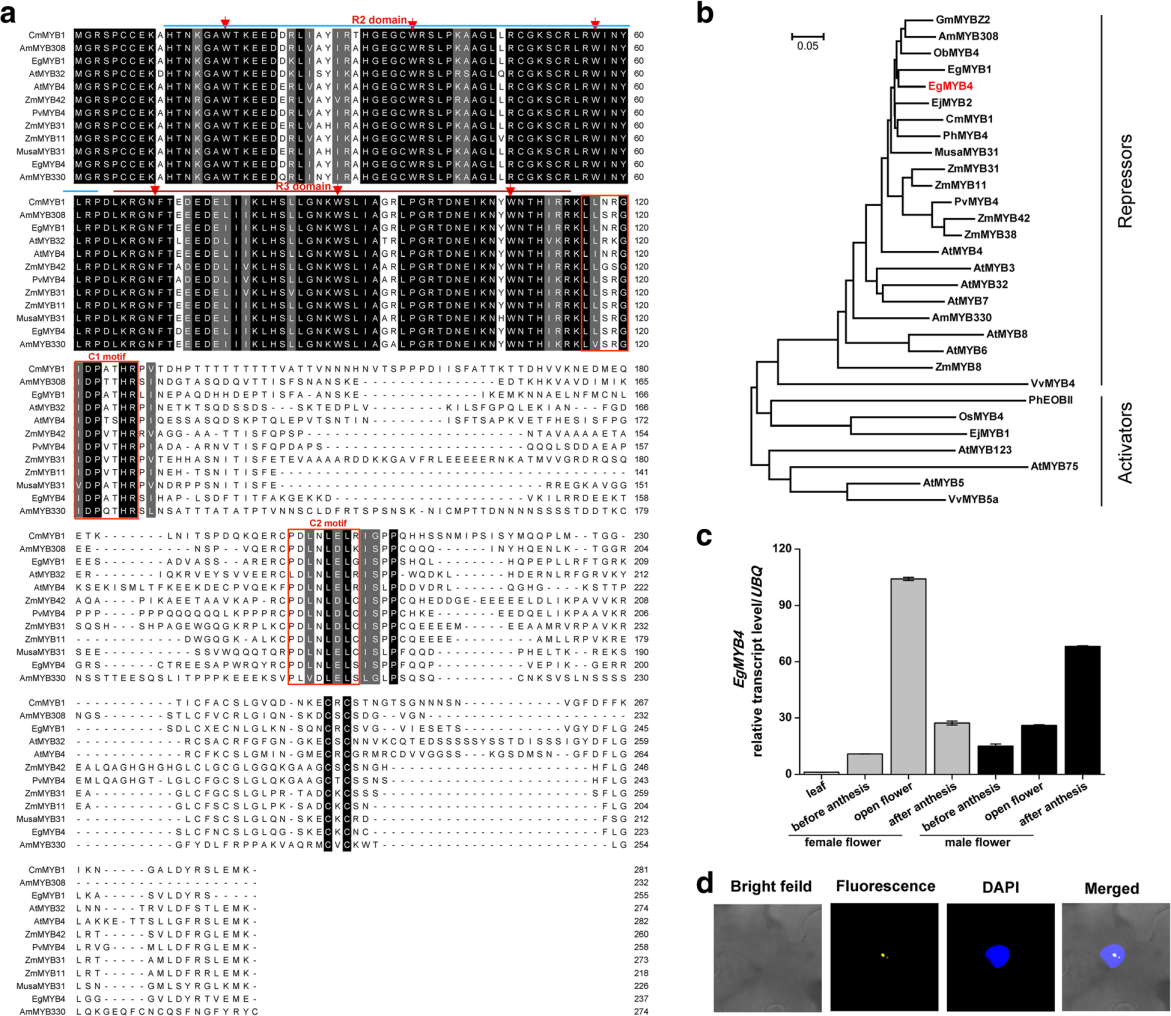
Characterization of EgMYB4. **a** The conserved amino acids, tryptophan (W) and phenylalanine (F) are indicated by arrows. The R2 and R3 domain is indicated by colored lines on top. The C1 (llsrGIDPxTHR) and C2 (pdLNL[D/E]L) motifs are highlighted with red boxes. **b** Phylogenetic relationships between EgMYB4 and other characterized R2R3-MYBs of subgroup 4. The tree was constructed using MEGA software. Amino acid sequences were used and bootstrap value was set to 1000 replicates. Selected protein accession numbers are listed in Additional file 5. **c** Transcript levels (± SE, *n* = 4) of *EgMYB4* in different tissues of oil palm. Samples from different floral stages were collected based on the description of Fig. 2b. Transcript levels were analyzed by qRT-PCR. *UBQ*: *Ubiquitin* gene. **d** Subcellular localization of EgMYB4. *N. benthamiana* leaves were transformed with *Agrobacterium* carrying either YFP or EgMYB4-YFP. After 48 h of incubation, the transformed cells were observed under a confocal microscope. Nuclei in leaf epidermal cells were stained with DAPI. Scale bar, 10 μM

Transcription factors (TFs) play an important role in regulating plant secondary metabolism [10, 42]. Seventy-nine upregulated and 219 downregulated TF transcripts were found in male and female open flowers when compared to leaves (Additional file 3A). The top 10 differentially expressed TFs between leaves and flowers are listed in Additional file 3B. Among them, the four MADS box TFs have been proposed to be involved in flower development [43]. The MYB-like TF was shown to be involved in regulating flowering time [44]. Out of the four MYB TFs identified, two were similar to *Arabidopsis MYB21* and one each to *Arabidopsis MYB108 and MYB4* respectively. Studies on *AtMYB21* showed that it functions in floral organ development particularly stamen development [45, 46], and *MYB108* was shown to regulate jasmonate-mediated stamen development [47]. The other differentially expressed MYB TF identified was *AtMYB4* which is implicated in regulating lignin pathway [20]. Analysis of *AtMYB4* and other R2R3-MYB belonging to subgroup 4 revealed that perturbations in lignin production was able to affect flux along the various phenylpropanoid pathways altering the formation of secondary metabolites [20, 26]. Since the production of both lignin and volatile phenylpropenes share common precursors of phenylpropanoid pathway, we decided to focus on this TF. Full length coding sequence of this MYB was obtained by reverse transcription PCR and named as *EgMYB4*. Amino acid sequence analysis indicated that it encodes a typical R2R3 domain and has an EAR motif (C2 motif) at the C-terminal similar to other members of R2R3-MYB subgroup 4 (Fig. 3a). Phylogenetic analysis based on amino acid sequences with other characterized R2R3-MYBs of subgroup 4 showed that EgMYB4 is closely related to *Eucalyptus gunnii* MYB1 (Fig. 3b). Expression profile analysis revealed high expression of *EgMYB4* in oil palm female and male open flowers when compared to leaves (Fig. 3c). Among the three developmental stages of flowers analyzed, EgMYB4 expression increased in open flowers as compared to before anthesis stage flowers correlating with the emission of methylchavicol. In female flowers, the expression of EgMYB4 decreased in after anthesis flowers while in male flowers it increased (Fig. 3c). To investigate the subcellular localization of EgMYB4, 35S: *EgMYB4*-*YFP* was transiently expressed in *N. benthamiana* leaves. Fluorescence analysis showed that EgMYB4 exclusively localize in the nuclear bodies as confirmed by 4, 6-diamidino-2-phenylindole (DAPI) staining (Fig. 3d).

### Phenylpropene synthases are enriched while *COMT* and *F5H* transcripts are repressed in oil palm flowers

**Fig. 4:**
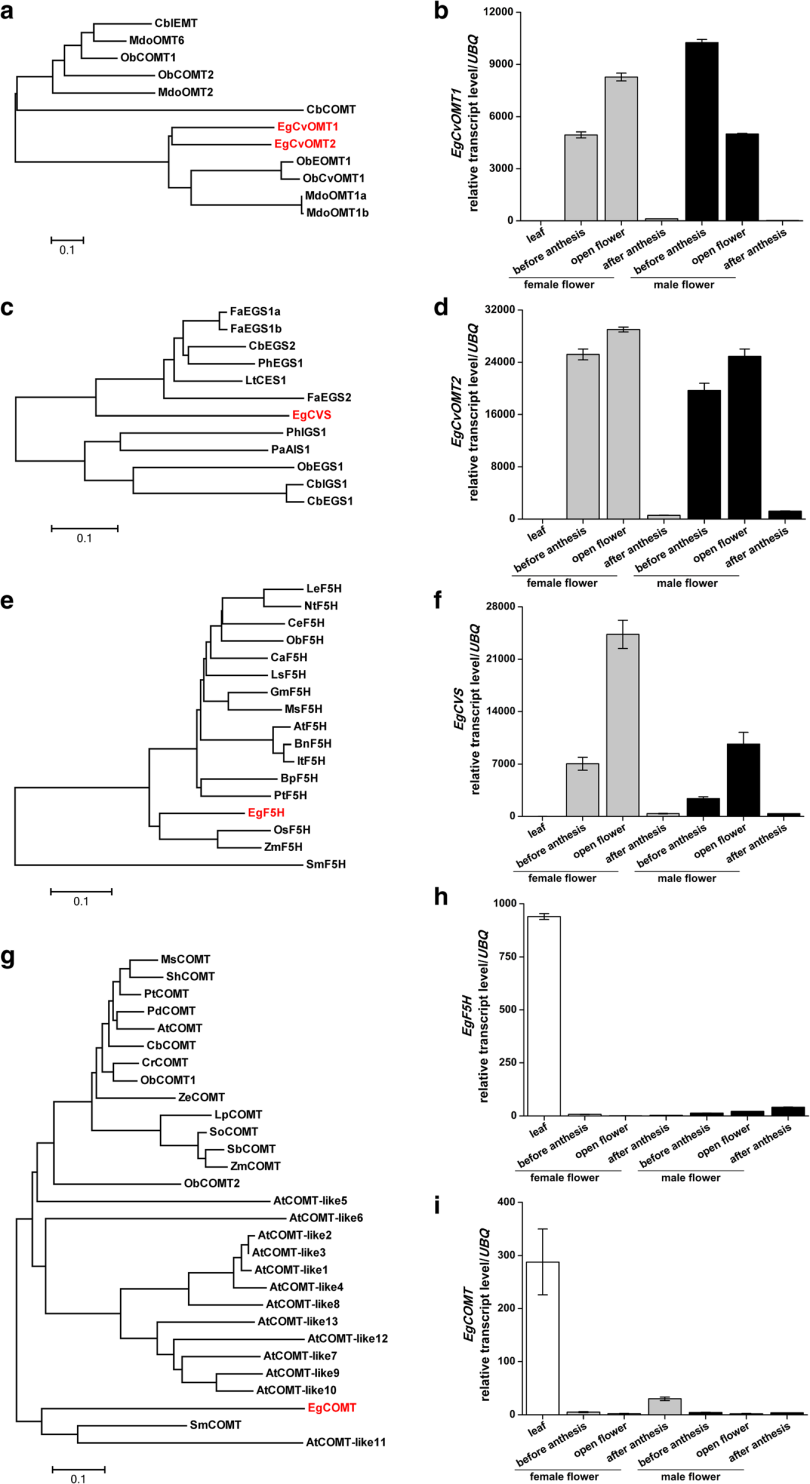
Expression of phenylpropanoid pathway genes in oil palm flower. Transcript levels (± SE, n = 4) of *EgCvOMT1* (**b**), *EgCvOMT2* (**d**), *EgCVS* (**f**), *EgF5H* (**h**) and *EgCOMT* (**i**) in different tissues of oil palm. Samples from different floral stages were collected based on the description of Fig. 2b. Transcript levels were analyzed by qRT-PCR. *UBQ*: *Ubiquitin* gene. **a** Phylogenetic relationships between EgCvOMT and O-methyltransferases from other plants. **c** Phylogenetic relationships between EgCVS and other NADPH dependent PIP reductases from plants. **e** Phylogenetic relationships between EgF5H and P450s from other plants. **g** Phylogenetic relationships between EgCOMT and O-methyltransferases from other plants. Neighbor-joining phylogenetic trees were constructed using MEGA5.1 based on multiple protein sequence alignments made with ClustalX

Among all the metabolic derivatives of phenylpropanoid pathway, lignin and phenylpropene biosynthesis share the most common precursors. To investigate the biosynthesis of lignin and methylchavicol in flowers, we first analyzed the expression of shared upstream biosynthesis genes from the RNA-Seq data. Many phenylpropanoid-pathway genes like *Phenylalanine ammonia-lyase* (*PAL*) *4*-*coumarate:CoA ligase* (*4CL*), *C4H*, *Cinnamoyl*-*CoA reductase* (*CCR1*) and *CAD* exist as a multigene family in various plant species. Studies have shown that they can have distinct or overlapping functions in phenylpropanoid metabolism depending on their substrate specificity [48]. We indeed identified more than one transcript encoding these genes from our RNA-Seq data of leaves and flowers. Few transcripts showed constitutive expression while few other transcripts had tissue specific expression (Additional file 4). These genes can act in different branches of phenylpropanoid biosynthesis pathway, hence their function in the pathway cannot be confirmed without the support of experimental data. To determine a clear expression pattern of the active metabolite pathway in flowers, genes specifically involved in latter steps of lignin and methylchavicol synthesis were investigated. Most differentially expressed transcripts between leaves and flowers were the genes encoding enzymes involved in the final two steps of methylchavicol biosynthesis; chavicol synthase (CVS) and chavicol O-methyltransferase (CvOMT). They were significantly up-regulated in both male and female open flowers (Fig. 4b–f). CVS belongs to PIP family of NADPH-dependent reductases which also include eugenol synthases (EGS) and isoeugenol synthases (IGS). EGS and IGS have been identified and characterized from basil, *Petunia*, *Clarkia*, anise and recently from strawberry [7, 49–52]. A chavicol synthase (LtCES1) was also characterized from *Larrea tridentata* that can act on both coniferyl acetate and coumaryl acetate substrates to form eugenol and chavicol, respectively [53]. The oil palm EgCVS showed high sequence similarity to these identified enzymes (Fig. 4c). Similarly, phenylpropene O-methyltransferases have been characterized from sweet basil and apple which are required for generating methylated phenylpropenes like, methyleugenol and methylchavicol [54, 55]. Two transcripts similar to chavicol O-methyltransferases (EgCvOMT1 and EgCvOMT2) were found in our RNA-Seq data, which shared high amino acid identity with sweet basil and apple CvOMTs (Fig. 4a). On the other hand, transcripts encoding genes involved in lignin biosynthesis genes such as *caffeic acid O*-*methyltransferase* (*COMT*) and *Ferulate*-*5*-*hydroxylase* (*F5H*) were highly down-regulated in oil palm male and female open flowers (Fig. 4h and i). *COMT* and *F5H* are key genes mainly committed to the formation of S-lignin subunit [12]. *EgCOMT* and *EgF5H* showed high similarity to the well-characterized genes from other species (Fig. 4e and g).Protein accession numbers and nucleotide sequences of the genes in Fig. 4 are listed out in Additional files 5 and 6 respectively.

### EgMYB4 interacts with *EgCOMT* promoter

**Fig. 5:**
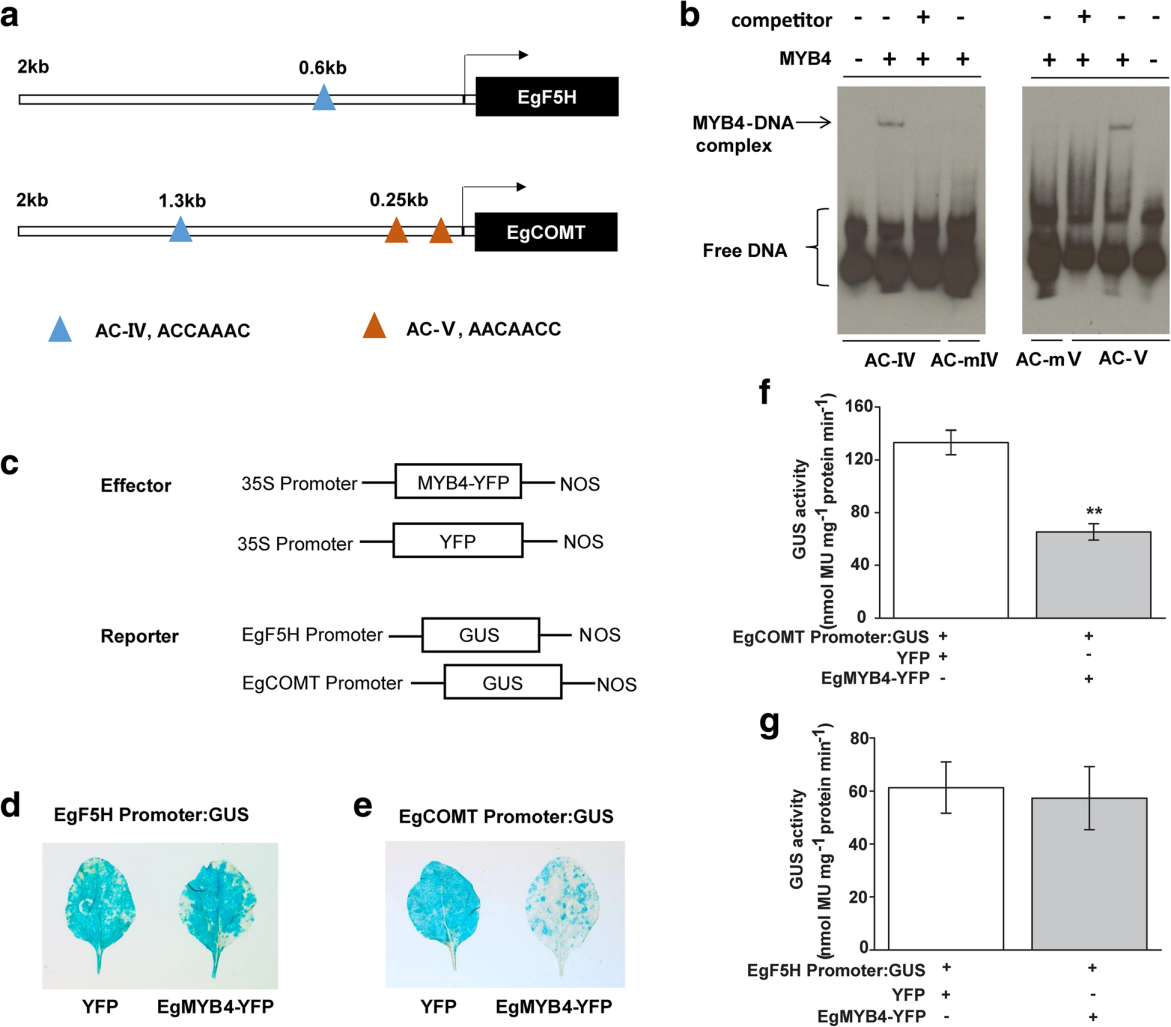
DNA binding and transcriptional repression activity of EgMYB4. **a** Schematic diagram of the *EgF5H* and *EgCOMT* promoter. The blue triangles represent AC-IV elements. The orange triangles represent AC-V elements. **b** DNA binding ability of EgMYB4 analyzed by electrophoretic mobility shift assay. The recombinant EgMYB4 protein can bind to the AC-IV sequence GAGGCCCATAAACCAAACGTAGAAAAG and AC-V sequence GTTATCCGTTCGCAACAACCCGCCATATCAACCAAG but not to the mutant AC-mIV sequence GAGGCCCATAAGAAGGGAGTAGAAAAG or AC-mV sequence GTTATCCGTTCGCGGAGGATCGCCATATCAACCAAG. Competition experiments were performed using unlabeled AC-IV and AC-V probes as competitors in a 250-fold molar excess. **c** Effectors and reporters used in this study. *EgF5H* promoter activity in *EgMYB4*-*YFP* expressing leaves and *YFP* expressing leaves was measured by GUS staining (**d**) and GUS quantification (**f**). *EgCOMT* promoter activity in *EgMYB4*-*YFP* expressing leaves and *YFP* expressing leaves was measured by GUS staining (**e**) and GUS quantification (**g**). Values are means ± SE (*n* = 8). Asterisks indicate significant differences in GUS activities between different treatments (**, *p* < 0.01; Student’s *t*-test)

AC elements are present in the promoters of many lignin biosynthesis genes and are known to be bound by MYBs which regulates their expression. Additionally, *COMT* and *F5H* genes are known to be down-regulated by EAR motif-containing MYBs in monocots. Maize ZmMYB11 was found to bind to the promoter of *ZmCOMT* in vivo; ZmMYB31 and ZmMYB42 was found to bind to the promoters of both *ZmCOMT* and *ZmF5H* genes and repress their expression [18, 26]. To investigate if *EgF5H* and *EgCOMT* are regulated by EgMYB4, we cloned and screened a 2 kb-promoter region of both genes. One AC-IV element (ACCAAAC) was found in both the promoters (Fig. 5a; [16]). Within 0.25 kb of promoter region of *EgCOMT*, we identified two new AC elements (AACAACC) and named it as AC-V. To test the interaction of EgMYB4 to these AC elements, an electrophoretic mobility shift assay (EMSA) was performed using the purified recombinant His-EgMYB4 protein. His-EgMYB4 bound to both AC- IV and AC-V elements but failed to interact with mutated versions of AC- IV (GAAGGGA) and AC-V (GGAGGAT) (Fig. 5b). DNA binding specificity was further confirmed by a competition experiment using 250-fold excess unlabeled cold probe which led to the disappearance of labeled DNA/protein complex.

Further, the transcriptional repression activity of EgMYB4 was determined by using *N. benthamiana* as a transient expression system [56]. Based on the distribution of AC elements, 1 kb promoter region of *EgF5H* and 2 kb promoter region of *EgCOMT* was introduced into pCAMBIA1391 vector. *EgF5H* promoter: *GUS* or *EgCOMT* promoter: *GUS* were used as reporters and 35S:*EgMYB4*-*YFP* was used as an effector (Fig. 5c). The promoter activity of *EgF5H* had no difference between *EgMYB4*-*YFP* and only *YFP* expressing leaves (Fig. 5d and g). However, the promoter activity of *EgCOMT* was significantly suppressed in *EgMYB4*-*YFP* expressing leaves when compared to leaves expressing only *YFP* (Fig. 5e and f). These results indicate that *EgCOMT* is repressed by EgMYB4. Suppression of COMT activity is known to reduce lignin formation [57–60]. From the above results, we can postulate that increased expression of *EgMYB4* in oil palm flowers reduces lignin synthesis by repressing *EgCOMT*, which might indirectly affect the metabolic flux into methylchavicol synthesis.

### Ectopic expression of *EgMYB4* in sweet basil decreases total lignin content and increases phenylpropene production

**Fig. 6:**
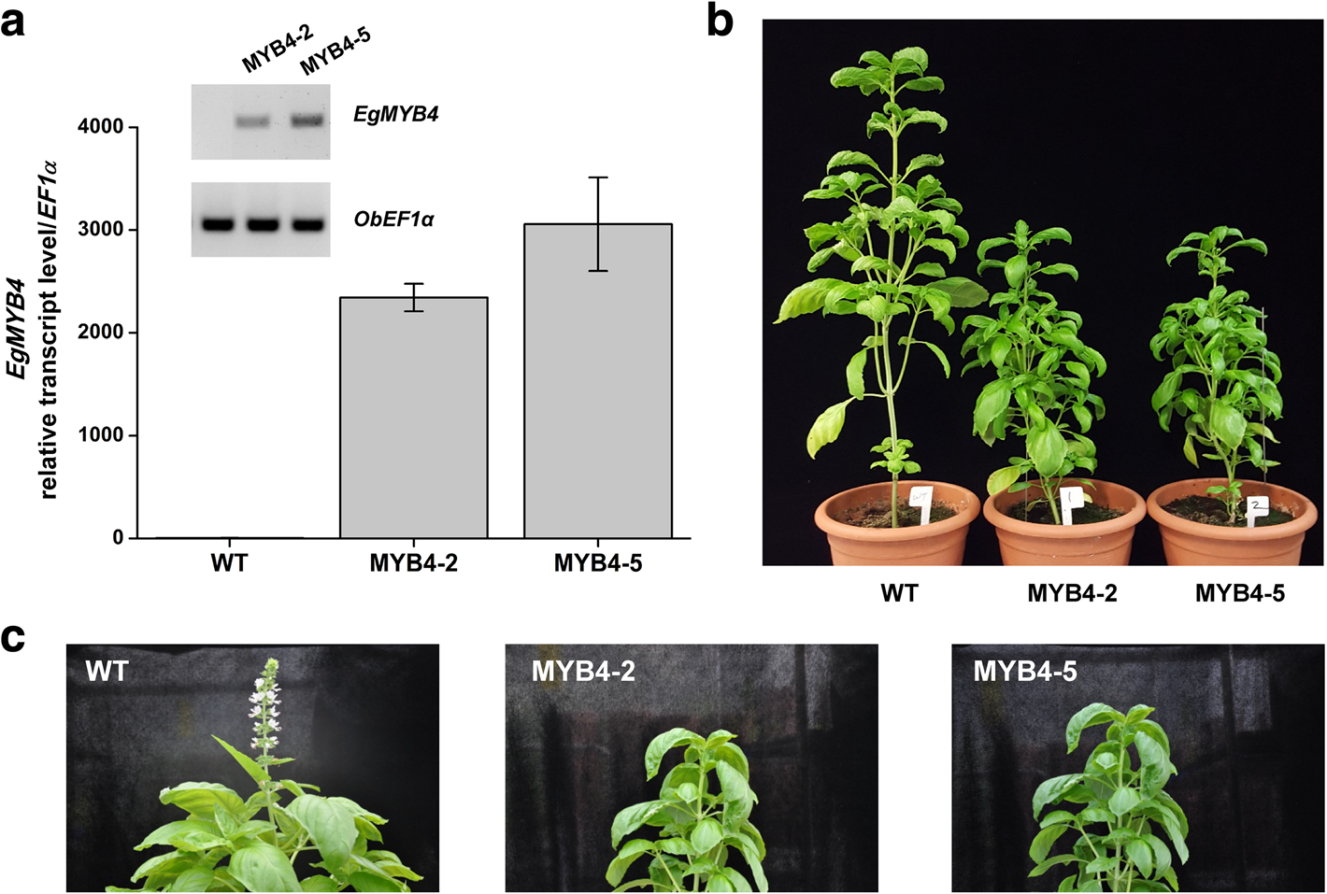
Transgenic sweet basil plants overexpressing *EgMYB4* were dwarfed, showed weak stem, small leaf size and delayed flowering. **a** Mean expression levels (± SE, *n* = 6) of *EgMYB4* in *EgMYB4* overexpressing sweet basil plants and wild type (WT) plants by semi-quantitative RT-PCR and qRT-PCR. *EF1α*: *elongation factor* gene. Growth phenotype of *EgMYB4* overexpressing sweet basil plants in seedling stage (**b**, six weeks after transferring to the soil) and reproductive stage (**c**, two months after transferring to the soil)

**Fig. 7:**
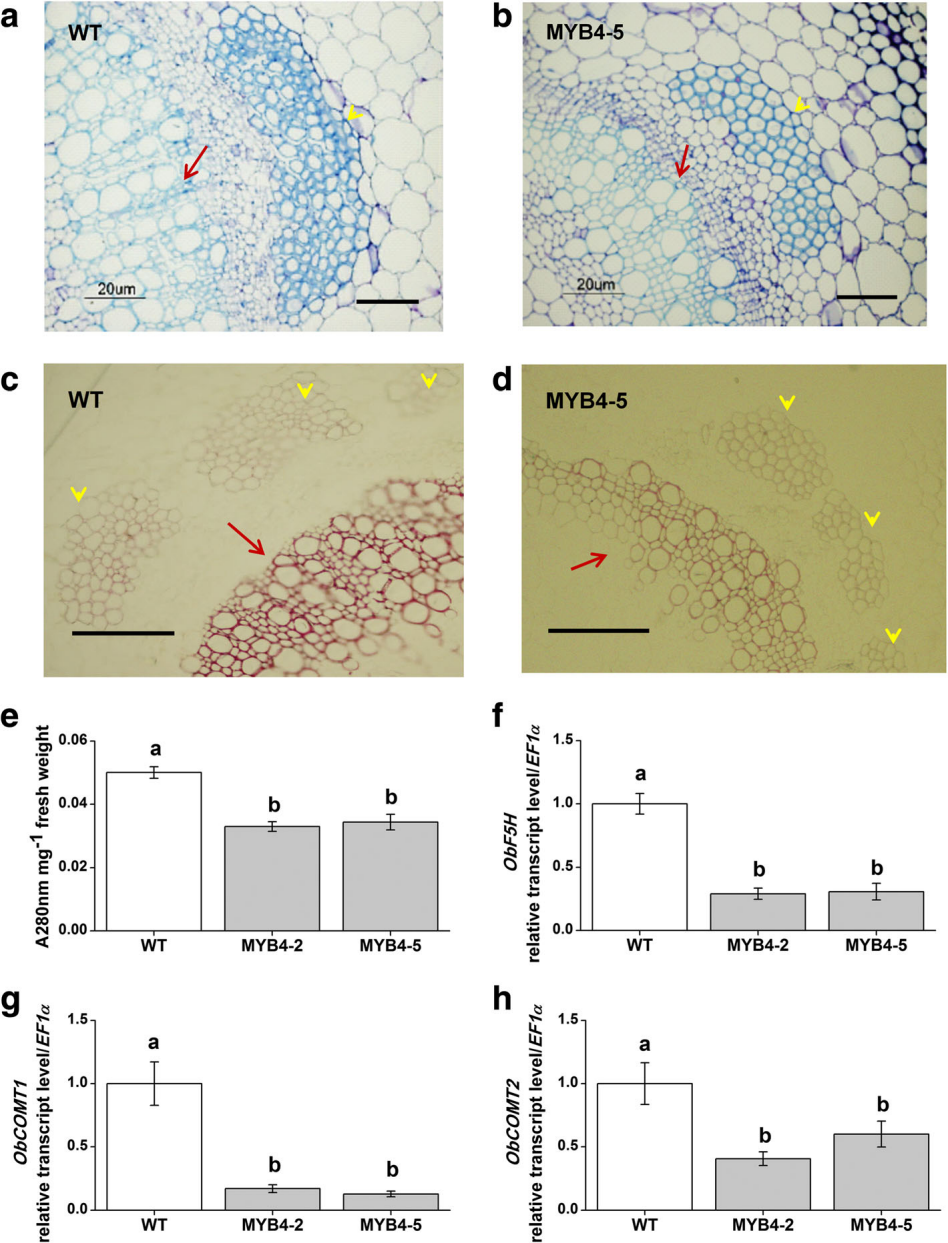
Transgenic sweet basil plants overexpressing *EgMYB4* show decreased lignin content. Transverse section of WT (**a**) and EgMYB4–5 line (**b**) stems stained with toluidine blue. Transverse section of WT (**c**) and EgMYB4–5 line (**d**) stems stained with phloroglucinol. Red arrows indicate the xylem tissue and yellow arrows indicate the sclerenchyma tissues. Scale bar, 20 μM. **e** Thioglycolic acid quantification of total lignin content in wild type (WT) and *EgMYB4*-expressing sweet basil plants. Values are mean ± SE (*n* = 6). Mean expression levels (± SE, n = 6) of *ObF5H* (**f**), *ObCOMT1* (**g**) and *ObCOMT2* (**h**) in *EgMYB4* overexpressing sweet basil plants and WT plants. Letters indicate significant differences among different lines (*p* < 0.05, Duncan’s multiple range test)

As oil palm is a non-model plant and transformation studies are not feasible, we decided to characterize EgMYB4 in sweet basil plants. Similar to oil palm flowers, sweet basil also produces phenylpropenes in leaf glandular trichomes which form the main components of sweet basil essential oil (Additional file 7; [9]). To validate our hypothesis that *EgMYB4* mediated lignin suppression in oil palm flowers affects flux into phenylpropene synthesis, *EgMYB4* was ectopically expressed in sweet basil plants. The sweet basil variety used in our study produces two types of phenylpropenes, majorly eugenol/methyleugenol and small amounts of methylchavicol. Five independent transgenic lines were initially selected and of them two lines, MYB4–2 and MYB4–5, were advanced for further characterization. Both of these lines showed high expression of *EgMYB4* transgene (Fig. 6a). Plants overexpressing *EgMYB4* showed many phenotypic alterations. These plants were dwarfed, exhibited weak stem, had reduced leaf size and delayed flowering (Fig. 6b and c). These phenotypes are characteristic of lignin-deficient mutants [26, 61]. In plants, the cell walls of sclerenchyma and vascular tissues are lignified providing strength and rigidity. Transverse sections of mature stems from transgenic and wild type (WT) sweet basil plants were analyzed by toluidine blue and phloroglucinol staining to look for possible changes in these lignified tissues. There was considerable amount of reduction in the number of lignified cell layers forming the sclerenchyma and vascular tissues in the transgenic plants (Fig. 7a-d). The amount of total lignin in mature stems was measured by thioglycolic acid method and was found to be reduced by 34.2% and 31.4% in *EgMYB4* overexpressing lines MYB4–2 and MYB4–5 respectively when compared to WT plants (Fig. 7e). These results indicate that overexpression of *EgMYB4* in sweet basil could reduce lignin biosynthesis in sweet basil plants.

We have performed RNA sequencing of sweet basil leaf glandular trichomes from which the orthologs of *F5H* and *COMT* genes were identified. Expression levels of these genes were measured in *EgMYB4* overexpressing and wild type sweet basil plants. The expression levels of *ObCOMT1*, *ObCOMT2* and *ObF5H* were significantly decreased in the transgenic lines when compared to WT plants (Fig. 7f-h). These results suggest that ectopic expression of EgMYB4 down regulates the expression of both *ObCOMT* and *ObF5H* genes thereby affecting lignin biosynthesis in sweet basil plants. Although in in vivo studies *EgMYB4* did not suppress *EgF5H,* we did observe a decrease in *ObF5H* expression when *EgMYB4* was ectopically expressed. The direct interaction of EgMYB4 with the promoters of *ObCOMTs* and *ObF5H* remains to be confirmed. However similar results have been observed with other R2R3-MYBs like *ZmMYB31* and *AtMYB4* where overexpression leads to dose dependent selection of additional target genes in transgenic plants [20, 26].

**Fig. 8:**
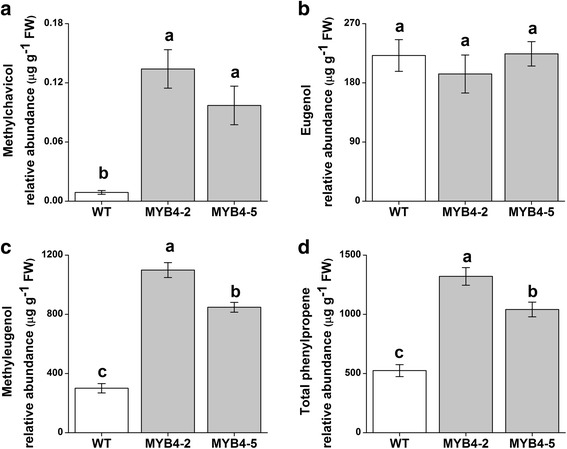
Transgenic sweet basil plants overexpressing *EgMYB4* show increased phenylpropenes biosynthesis. Mean levels (± SE, *n* = 6) of methylchavicol (**a**), eugenol (**b**), methyl eugenol (**c**) and total phenylpropenes (**d**) produced by wild type (WT) and EgMYB4-expressing sweet basil plants. Letters indicate significant differences among different lines (*p* < 0.05, Duncan’s multiple range test)

To determine the effect of *EgMYB4* overexpression on phenylpropenes, volatile compounds were extracted and analyzed from transgenic lines and WT plants. The transgenic plants exhibited a significant increase in total phenylpropene levels (Fig. 8d). The amount of methylchavicol in MYB4–2 and MYB4–5 was ~15- and 10-folds higher than WT plants (Fig. 8a). Meanwhile, the amount of methyleugenol in MYB4–2 and MYB4–5 was ~ 4- and 3-folds higher than WT plants (Fig. 8c). However, we did not see any changes in eugenol levels (Fig. 8b). This result suggests that ectopic expression of EgMYB4 can affect flux into phenylpropene pathway.

## Discussion

Efficient pollination is vital for plant’s survival. The interaction between plant and its pollinator is considered to be mutualistic and the result of coevolution between plant and pollinator species. A stringent one-to-one coevolution is generally found between one plant species and its insect partner, for example fig plants and its specific pollinator [62]. In case of oil palm, average yields in Southeast Asia increased considerably only after introduction of *Elaedobius kamarunicus* from Africa. This strongly suggests that oil palm is highly dependent on just a single species of insect for pollination suggesting a specialized mutualistic palm–pollinator relationship [63]. Phenylpropenes form one of the main components of floral scents that help attract pollinators. Oil palm flowers emit methylchavicol to attract insect pollinators. Phenylpropenes are derived from the general phenylpropanoid pathway. Pathway studies propose that both lignin and phenylpropene production share common substrates [13]. Hence within a cell, tight regulation of metabolic pathway enzymes is required to ensure that substrates used by competing pathways are regulated aptly to produce the desired metabolic outcome. The biosynthesis of lignin consumes large amounts of carbon resources. The biomass of the oil palm is lignocellulosic rich with lignin contents ranging from 18 to 23% among different varieties and tissues [64]. From a plant’s perspective, suppression of lignin biosynthesis in flowers allows the plant to reallocate carbon sources towards a vital floral phenylpropene production, which will aid in attracting pollinators. Transcriptome data analysis of open oil palm flowers revealed that *EgCVS* and *EgCvOMT* are highly expressed in flowers whereas expression levels of *EgCOMT* and *EgF5H* are significantly reduced when compared to leaves (Additional file 4). By comparative transcriptome analysis and protein-DNA interaction assays, we identified a R2R3 EAR-motif-containing EgMYB4 from oil palm which can bind to AC elements of *EgCOMT* and suppress its activity in vivo*.* No interaction with *EgF5H* was observed (Fig. 5). Although one AC element exists in the promoter of *EgF5H* which was cloned, we cannot completely rule out the possibility that the length of the promoter tested might not have been enough to observe the binding of EgMYB4 to *EgF5H*. Additionally, there might be other EAR-motif-containing MYBs that represses *EgF5H* in oil palm flowers. Monocot transcription factors like ZmMYB11, MusaMYB31, ZmMYB31, ZmMYB42 and PvMYB4 which show high sequence identity with EgMYB4 are reported to repress COMT along with other genes in the lignin biosynthesis pathway [25–28]. In our study, we tested the interaction of EgMYB4 with the promoters of *EgCOMT* and *EgF5H* only, we cannot dismiss the possibility that it might regulate other genes in phenylpropanoid pathway. qRT-PCR analysis of *EgMYB4* at different developmental stages of flowers showed high levels of *EgMYB4* in after anthesis stage male flowers. At this stage, flowers do not emit phenylpropenes suggesting additional role for *EgMYB4* in flowers. Perturbation in one branch of phenylpropanoid pathway is known to affect other branches as well. After examining the transcripts of genes involved in flavonoid pathway from the RNA-Seq data of oil palm flowers, we were not able to observe a clear pattern that would suggest either up or downregulation of flavonoid pathway in flowers (Additional file 8). Further work is required to analyze other phenylpropanoid derived metabolites in oil palm flowers apart from methylchavicol to determine the effect of *EgMYB4* on their production.

Overexpression of *EgMYB4* in sweet basil resulted in increased phenylpropene levels and decreased lignin content (Figs. 7 and 8). Sweet basil *EgMYB4* overexpressing plants exhibited growth phenotypes typical to lignin deficient plants, including dwarfism, weakened stem, reduced leaf size and delayed flowering [26]. These phenotypes of decreased lignin imply that ectopic expression of *EgMYB4* can affect lignin biosynthesis genes in sweet basil. Downregulation of *ObCOMTs* and *ObF5H* was observed in transgenic plants. However, in the in vivo studies EgMYB4 did not suppress *EgF5H*. There is a possibility that EgMYB4 might have different targets in different plants or the resulting perturbation in the lignin pathway might affect the steady state mRNA level of *ObF5H*. Additionally, this might also be due to the high level of *EgMYB4* expression in the transgenics. Similar results have been observed with *AtMYB4* and *ZmMYB31*. AtMYB4 mainly regulates cinnamate 4-hydroxylase (*C4H*) to control hydroxycinnamic acid metabolism. But when overexpressed it was able to down regulate the expression of additional genes (*CHS*, *4CL1* and *4CL3*) in the pathway. It was found that there is a dose dependent selection of target genes by AtMYB4 [20]. Similarly, ZmMYB31 interacts with *ZmCOMT* and *ZmF5H* promoters in vivo. However, it can repress the expression of Arabidopsis *4CL* and *C3H* when overexpressed in Arabidopsis [26]. The direct binding of EgMYB4 to the promoters of sweet basil *COMTs, F5H* and other lignin pathway genes needs to be confirmed. Downregulation of *COMT* and *F5H* by transgenic approaches has been reported in many plants like Arabidopsis, tobacco, poplar, alfalfa, maize, switchgrass and fescue to manipulate lignin polymer. They mainly affect the formation of S-lignin subunits [57–60, 65–69]. We observed that both methylchavicol and methyleugenol was increased in *EgMYB4* overexpressing sweet basil lines which are derived from p-coumaryl and coniferyl alcohol respectively. Probably the downregulation of sinapyl alcohol (S-lignin) pathway increases flux towards the formation of *p*-coumaryl and coniferyl alcohol thus enhancing the formation of both methylchavicol and methyleugenol which are derived from these precursors in sweet basil.

In oil palm, transcripts encoding enzymes directly responsible for methylchavicol biosynthesis like EgCvOMT and EgCVS show transcriptional activation only in flowers (Additional file 4). However, in addition to direct activation of enzymes that are responsible for metabolite production, activation and suppression of enzymes involved in flux leading to its formation also help to fine tune the amount of metabolite formation, in a particular tissue at a developmentally relevant time**.** For example, in Petunia R2R3-MYBs *ODORANT1* (*ODO1*), *EMISSION OF BENZENOIDS I, EMISSION OF BENZENOIDS II* (*EOBII*), are all identified as positive regulators of various genes involved in the production of floral volatiles [29–33]. But *PhMYB4* belonging to R2R3-MYB subgroup 4 acts as a repressor of *C4H* gene of phenylpropanoid pathway and indirectly affects the formation of petunia floral volatiles [33]. Similarly, production of methylchavicol in oil palm flowers can be regulated at various levels by different genes acting as positive or negative regulators. Multiple or single TFs might be involved in stage specific activation of EgCvOMT and EgCVS. Our work presented here shows the regulation imparted by one of the probably many transcription factors involved in temporal and spatial regulation of methyl chavicol biosynthesis in oil palm flowers. Emission of methylchavicol forms an important agronomic trait in oil palm as it affects pollination. In our study, we identified a transcription factor potentially regulating methylchavicol emissions and enzymes catalyzing its production. These genes may be used as potential candidates in molecular breeding strategies of oil palm. Discovering genetic variations of these genes in different oil palm accessions and its effect on emission/yield can help design breeding of high yielding varieties.

## Conclusions

A volatile phenylpropene, methylchavicol is released from oil palm flowers to attract the pollinator weevil. RNA-Seq analysis of flowers showed increased expression of transcripts coding for enzymes involved in methylchavicol production but decreased expression of *COMT* and *F5H* transcripts which are specifically involved in lignin formation. We identified a R2R3-MYB enriched in flowers, *EgMYB4* that can bind to *EgCOMT* promoter and suppress it. Functional analysis of *EgMYB4* in sweet basil demonstrated the ability of *EgMYB4* to reduce lignin formation and enhance the formation of phenylpropene suggesting a similar role for *EgMYB4* in oil palm flowers. This study on methylchavicol emission in oil palm flowers will further enrich our understanding of transcriptional regulation of the complex phenylpropanoid pathways which produces an array of compounds in plants. Moreover, it will help in oil palm breeding and metabolic engineering of phenylpropanoid pathway to produce aromatic compounds of interest.

## Methods

### Plant materials

The oil palm species used in this study was *Dura* [2]. Samples from different flower stages and leaves were collected from palm trees planted in Temasek Life Sciences Laboratory, Singapore and Wilmar International Plantation, Palembang, Indonesia. Commercial sweet basil (*O. basilicum*) was tested for its secondary metabolites by GC-MS and grown in green house under natural light conditions. *Agrobacterium* mediated transformation of sweet basil was performed as previously described by [70]. T_0_ and T_1_ transgenic plants were selected using GFP as visual marker. For all experiments, T_1_ plants were used. *Nicotiana benthamiana* seeds were germinated on MS plate and transferred into soil. Twenty days after growing in the greenhouse, the seedlings were used for Agro-infiltration.

### Compound analysis

1.5 cm of one oil palm male flower bunch or 3 female flowers were placed in a 5 mL glass bottle containing 2 mL hexane and shaken for 10 min at room temperature. After centrifugation, 500 mL of supernatant was transferred into a 2 mL GC vial and analyzed by gas chromatography-mass spectrometry (GC-MS). For sweet basil samples, leaves were ground in liquid nitrogen and 200 mg of powder was transferred to 1.5 mL eppendorf tubes. 500 μL of hexane (containing 20 μg of diethyl sebacate as internal standard) was added and vortexed for 2 min. After centrifugation, 400 μL of supernatant was transferred into a 2 mL GC vial and analyzed by GC-MS. Six plants from each transgenic line were measured.

### RNA isolation and RNA sequencing

RNA from oil palm tissues was extracted as described previously [71]. Briefly, about 0.5 g of each sample was ground in liquid nitrogen and the powder was then transferred to a pre-chilled polypropylene (Falcon) tube. 5 mL of pre-heated (65 °C) CTAB extraction buffer (2% (*w*/*v*) CTAB, 2% (w/v) polyvinylpyrrolidone (PVP-40), 100 mM Tris-HCl (pH 8.0), 25 mM EDTA, 2 M NaCl, 0.1% spermidine and 2% β-mercaptoethanol) was added to each tube and samples were incubated for 30 min at 65 °C. Later, the samples were extracted with chloroform: isoamylalcohol (24:1) for 2 times. The supernatant (1.0 mL) was then transferred to RNase free1.5 mL eppendorf tubes and 0.5 mL of 96–100% ethanol was added. The mixture was immediately loaded onto RNA binding columns (Qiagen RNA Mini extraction kit) and RNA was extracted according to manufacturer’s protocol. RNA sequencing and assembly was performed as described previously [72]. RNA from sweet basil samples were isolated by using RNA Mini extraction kit (Qiagen).

### Quantitative real-time PCR (qRT-PCR) and reverse transcription PCR (RT-PCR)

Eight hundred ng of total RNA for each sample was reverse transcribed using the PrimeScript™ RT-PCR Kit (TaKaRa). qRT-PCR was performed on an ABI 7900 HT fast real time system (Life technologies) using SYBR Green Real-time PCR Master Mixes (Life technologies). For RT-PCR, genes were amplified in T100™ Thermal Cycler (Bio-Rad) by the following program, 95 °C for 2 min; 23 cycles of 95 °C for 40s, 60 °C for 40s, 72 °C for 15 s; 72 °C for 5 min. 10 μL of PCR products were analyzed by gel electrophoresis. The primers used for RNA detection of target genes by qRT-PCR and RT-PCR are listed in Additional file 9. Oil palm *Ubiquitin* gene (*EgUBQ*) or sweet basil *elongation factor* (*ObEF1α*) gene was used as internal controls.

### Constructs

Full-length open reading frames encoding EgMYB4 without a stop codon were amplified by PCR using *Pfu* DNA polymerase (Thermo Scientific) with primers listed in Additional file 9. The obtained DNA fragments were cloned either into pBA-YFP vector [56] to generate YFP fused protein or pET28b (Novagene) to generate His-tag fused protein.

### Purification of recombinant protein

The construct containing His-MYB4 was transformed into *E*.*coli* BL21 (DE3). Expression of His-MYB4 was induced by adding 0.4 mM isopropyl-β-thiogalactopyranoside (IPTG) followed by an incubation at 37 °C for 3 h. Later, the cells were collected and the recombinant protein was purified using His-Trap (GE healthcare) according to the manufacturer’s instruction*.*

### Electrophoretic mobility shift assay (EMSA)

The probes used in EMSA were all labelled by Biotin and are listed in Additional file 9. EMSA was performed using a LightShift Chemiluminescent EMSA Kit (Thermo) according to the manufacturer’s instructions. Competition experiments were performed using unlabeled DNA as a competitor in a 250-fold molar excess.

### Transcriptional repression assay

The 2 kb promoter region of *EgCOMT* and 1 kb promoter region of *EgF5H* were PCR-amplified (primers are listed in Additional file 9) and cloned into pCAMBIA1391. All constructs were introduced into AGL1 *Agrobacterium*. Leaves of *N. benthamiana* were agroinfiltrated with the indicated constructs (Fig. 5) at a ratio of 1:1. Two days after infiltration leaves were harvested and frozen in liquid nitrogen or stained with GUS staining buffer [56]. Each treatment was repeated eight times. GUS quantitative assay was performed as described previously [56].

### Lignin measurement

Total lignin content was measured using thioglycolic acid method as described previously [61]. 100 mg of fresh sweet basil stems were used and six plants from each transgenic line were measured.

### Histology

Stems from the basal portion of adult wild type sweet basil and transgenic plants were fixed in historesin and sectioned. Sections were stained either with toluidine blue or phloroglucinol for lignin analysis. Lignin analysis was performed as described previously [73].

### Data analysis

Differences in total lignin and phenylpropene levels on different lines were determined by analysis of variance (ANOVA). Differences in GUS activities on different treatments were analyzed by using the Student’s t-test. All tests were carried out with Statistica (Statistica, SAS Institute Inc., http://www.sas.com/).

## Additional files

Additional file 1: Analysis of volatile compounds in different oil palm tissues. (DOCX 115 kb)

Additional file 2: Overview of RNA-seq result. (DOCX 302 kb)

Additional file 3 Transcription factors found in RNA-seq data. (DOCX 209 kb)

Additional file 4: Heat map of phenylpropanoid pathway transcripts expression in oil palm leaves and male and female open flowers generated by Heatmapper. (DOCX 490 kb)

Additional file 5: List of plant genes used in the phylogenetic analysis described in Figs. 3 and 4. (DOCX 25 kb)

Additional file 6: Nucleotide sequence information of phenylpropanoid pathway genes from RNA-seq. (DOCX 22 kb)

Additional file 7: GC-MS analysis of phenylpropenes in sweet basil leaves. (DOCX 74 kb)

Additional file 8: Expression levels of predicted flavonoid biosynthesis genes in oil palm leaves and flowers. (DOCX 20 kb)

Additional file 9: DNA primers used in this study. (DOCX 21 kb)

## Abbreviations

C4H: cinnamate 4-hydroxylase
*CAD*: cinnamyl alcohol dehydrogenase
*CCR*: cinnamoyl-CoA reductase
COMT: caffeic acid O-methyltransferase
EMSA: electrophoretic mobility shift assay
GC-MS: gas chromatography-mass spectrometry
GFP: green fluorescent protein
GUS: β-glucuronidase
RNA-Seq: RNA sequencing
TF: transcription factors
YFP: yellow fluorescent protein
