# Supplementary material for: Comparative transcriptome analysis of oil palm flowers reveals an EAR-motif-containing R2R3-MYB that modulates phenylpropene biosynthesis
Source: BMC Plant Biol. 2017 Nov 23;17:219. doi: 10.1186/s12870-017-1174-4 (PMC5701422; doi:10.1186/s12870-017-1174-4)
Supplement: Supplementary file 1 — Analysis of volatile compounds in different oil palm tissues. (DOCX 115 kb) [file 12870_2017_1174_MOESM1_ESM.docx]

**
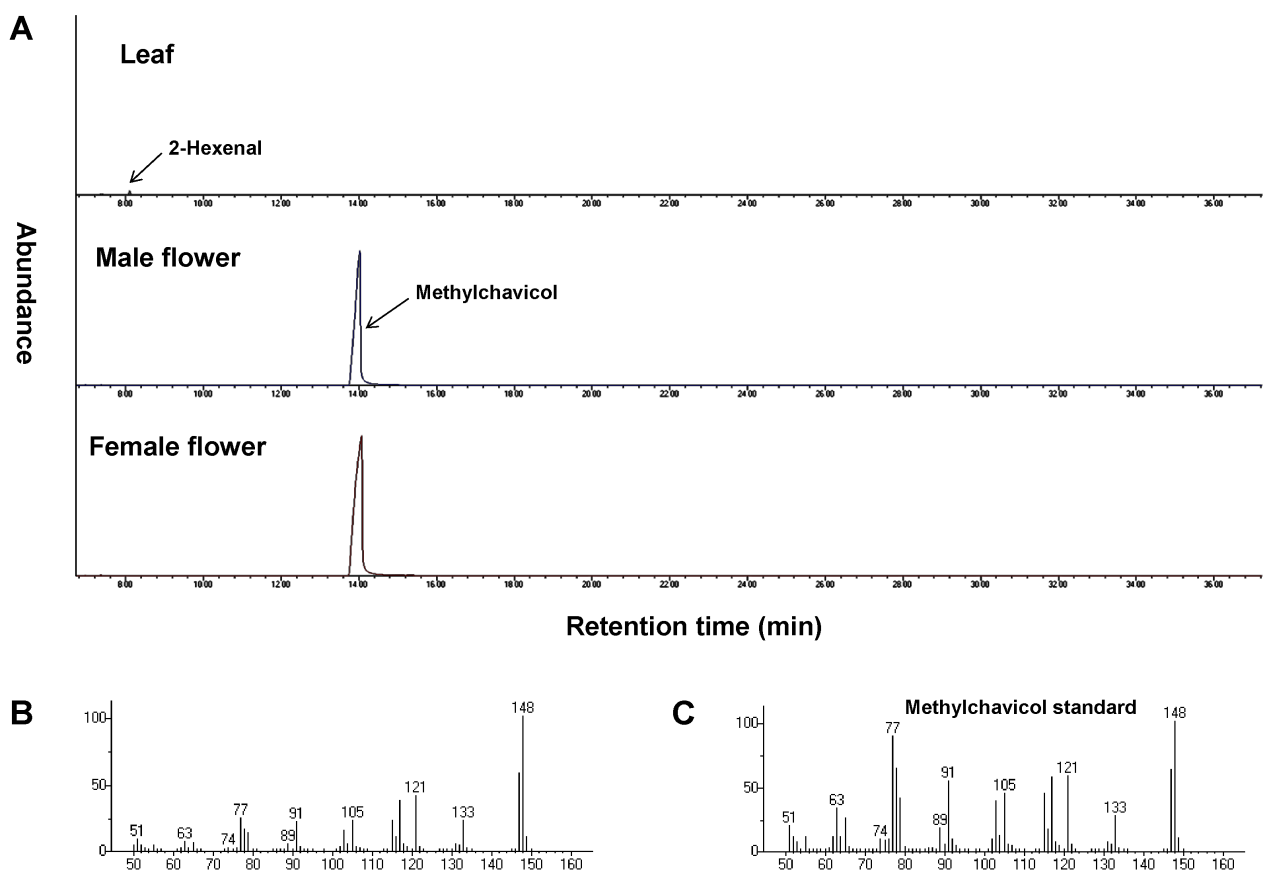
**

**Additional file 1.** Analysis of volatile compounds in different oil palm tissues.

(A) GC-MS analysis of volatile compounds in oil palm leaves and flowers. (B) Mass spectra of the compound produced by male and female oil palm flowers at open stage. (C) Mass spectra of methylchavicol standard.
