# Supplementary material for: Comparative transcriptome analysis of oil palm flowers reveals an EAR-motif-containing R2R3-MYB that modulates phenylpropene biosynthesis
Source: BMC Plant Biol. 2017 Nov 23;17:219. doi: 10.1186/s12870-017-1174-4 (PMC5701422; doi:10.1186/s12870-017-1174-4)
Supplement: Supplementary file 2 — Overview of RNA-seq result. (DOCX 302 kb) [file 12870_2017_1174_MOESM2_ESM.docx]

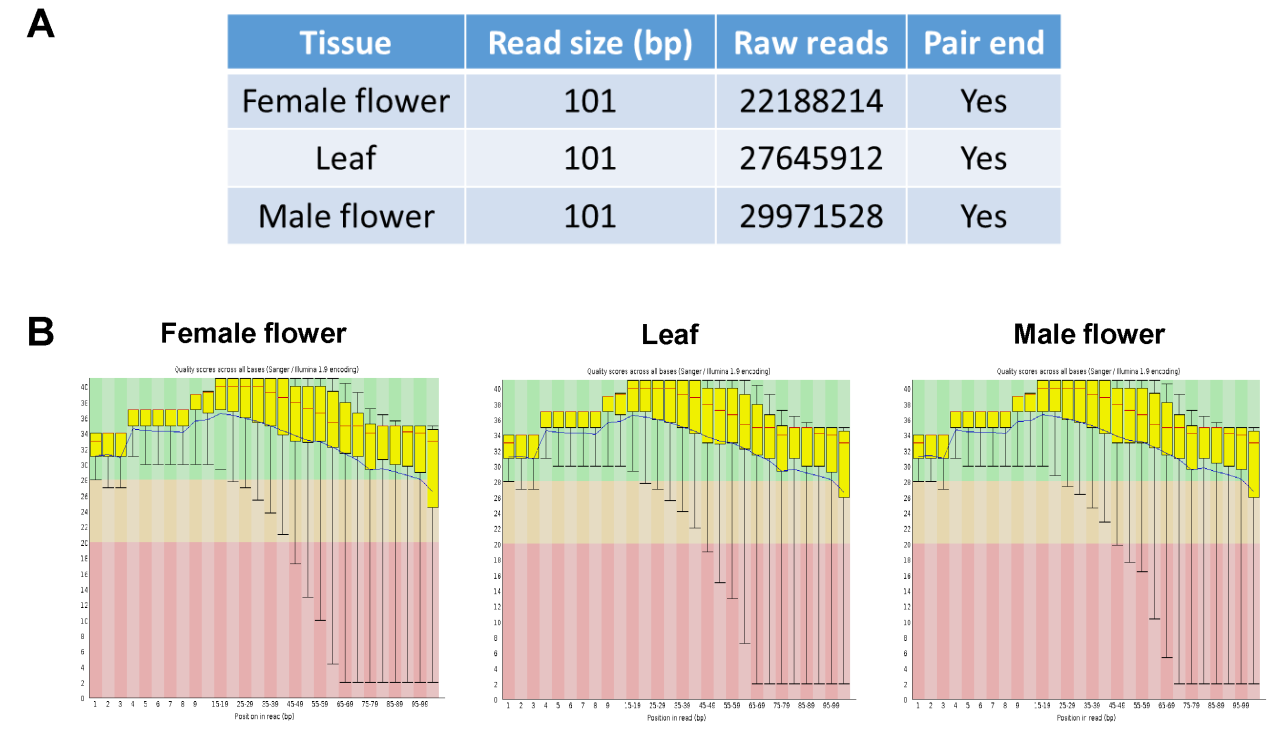


**Additional file 2.** Overview of RNA-seq result.

(A) Basic statistics for the sequencing results of three tissues. (B) Quality for raw sequencing results. The sequence quality was evaluated by FastQC. X-axis, position of 101 bp reads; Y-axis, score for each position.
