# Supplementary material for: Comparative transcriptome analysis of oil palm flowers reveals an EAR-motif-containing R2R3-MYB that modulates phenylpropene biosynthesis
Source: BMC Plant Biol. 2017 Nov 23;17:219. doi: 10.1186/s12870-017-1174-4 (PMC5701422; doi:10.1186/s12870-017-1174-4)
Supplement: Supplementary file 3 — Transcription factors found in RNA-seq data. (DOCX 209 kb) [file 12870_2017_1174_MOESM3_ESM.docx]

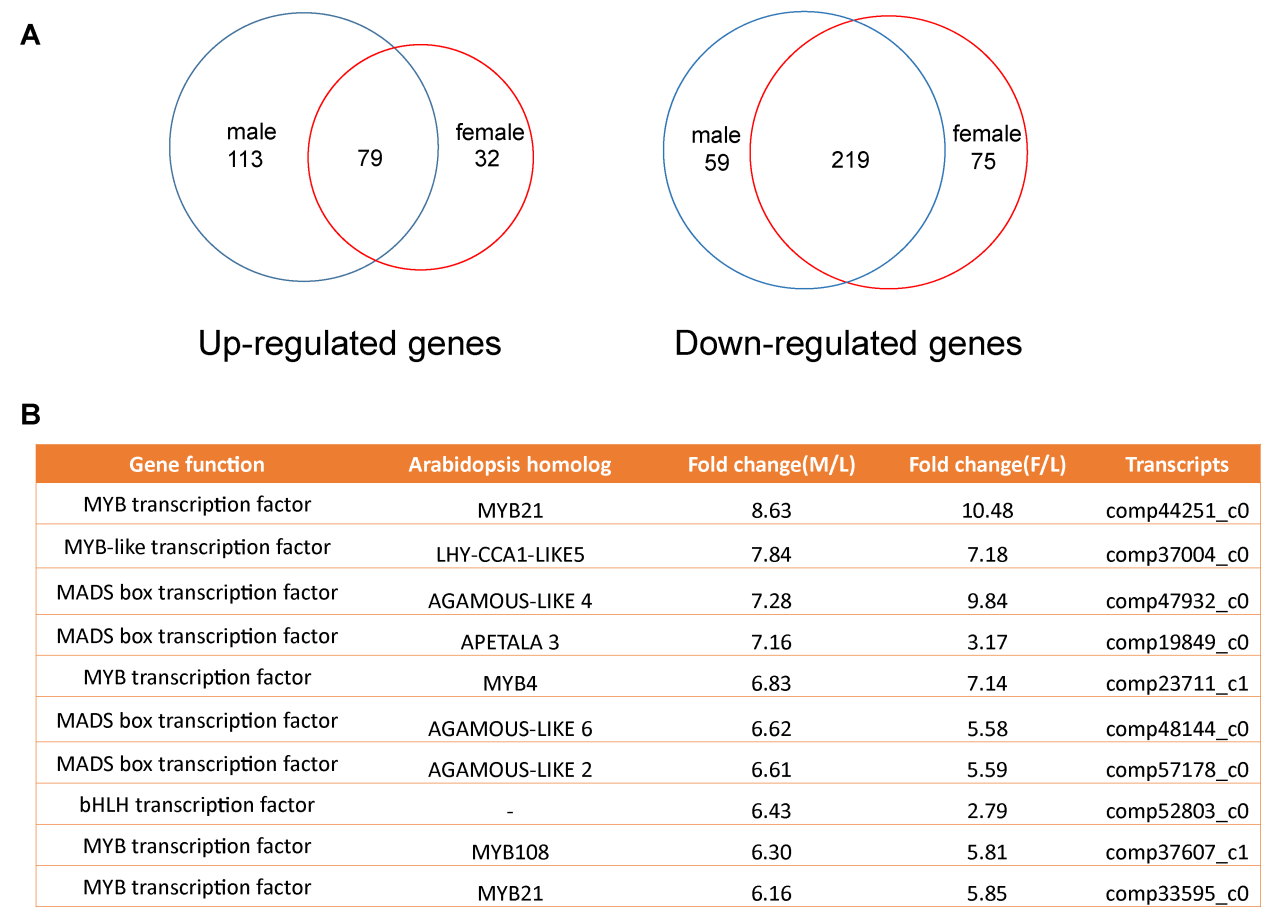


**Additional file 3.** Transcription factors found in RNA-seq data.

1. Venn diagram of differentially expressed isotigs in male and female flowers. (B)

Top 10 up-regulated transcription factors in oil palm flower.
