# Supplementary material for: Comparative transcriptome analysis of oil palm flowers reveals an EAR-motif-containing R2R3-MYB that modulates phenylpropene biosynthesis
Source: BMC Plant Biol. 2017 Nov 23;17:219. doi: 10.1186/s12870-017-1174-4 (PMC5701422; doi:10.1186/s12870-017-1174-4)
Supplement: Supplementary file 4 — Heat map of phenylpropanoid pathway transcripts expression in oil palm leaves and male and female open flowers generated by Heatmapper. (DOCX 490 kb) [file 12870_2017_1174_MOESM4_ESM.docx]

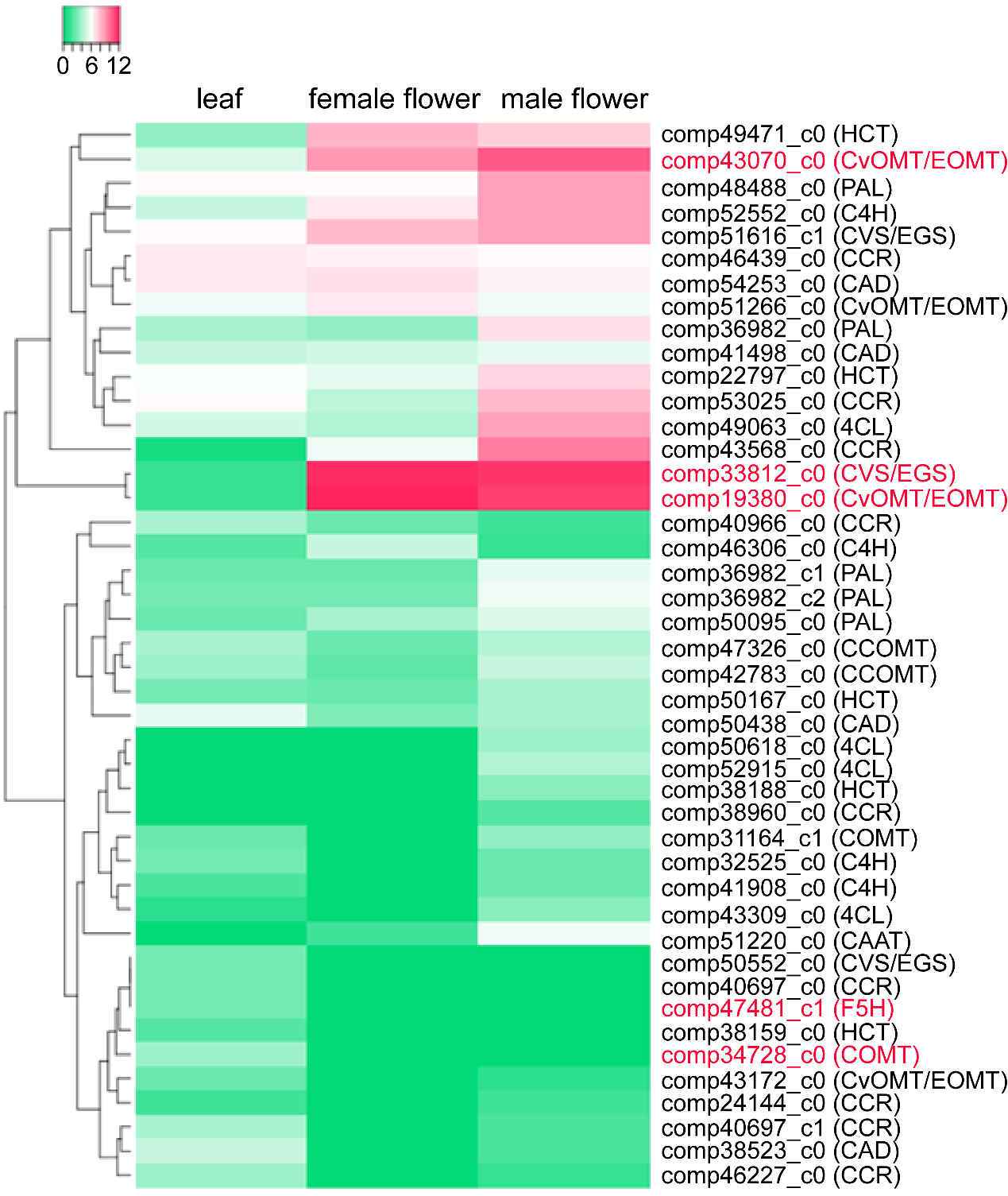


**Additional file 4.** Heat map of phenylpropanoid pathway transcripts expression in oil palm leaves and male and female open flowers generated by Heatmapper.

The details of gene names are listed in Figure 1. The color gradient represents the log2 normalized expression map. Genes analyzed in this study are highlighted in red.
