# Supplementary material for: Comparative transcriptome analysis of oil palm flowers reveals an EAR-motif-containing R2R3-MYB that modulates phenylpropene biosynthesis
Source: BMC Plant Biol. 2017 Nov 23;17:219. doi: 10.1186/s12870-017-1174-4 (PMC5701422; doi:10.1186/s12870-017-1174-4)
Supplement: Supplementary file 5 — List of plant genes used in the phylogenetic analysis described in Figs. 3 and 4. (DOCX 25 kb) [file 12870_2017_1174_MOESM5_ESM.docx]

**Additional file 5.** List of plant genes used in the phylogenetic analysis described in Figure 3 and 4.

| **Name** | **Species** | **Genebank accession no.** |
| --- | --- | --- |
| ObEGS1 | *Ocimum basillcum* | DQ372812 |
| PhIGS1 | *Petunia x hybrida* | DQ372813 |
| LtCES1 | *Larrea tridentata* | KF543262 |
| CbIGS1 | *Clarkia breweri* | EF467238 |
| CbEGS1 | *Clarkia breweri* | EF467239 |
| CbEGS2 | *Clarkia breweri* | EF467240 |
| PhEGS1 | *Petunia x hybrida* | EF467241 |
| PaAIS1 | *Pimpinella anisum* | EU925388 |
| FaEGS1a | *Fragaria x ananassa* | KF562264 |
| FaEGS1b | *Fragaria x ananassa* | KF562265 |
| FaEGS2 | *Fragaria x ananassa* | KF562266 |
| ZeCOMT | *Zinnia elegans* | Q43239 |
| PdCOMT | *Populus dulcis* | Q43609 |
| PtCOMT | *Populus tomentosa* | AAF63200 |
| ShCOMT | *Stylosanthes humilis* | 2119166A |
| CbCOMT | *Clarkia breweri* | AAB71141 |
| AtCOMT | *Arabidopsis thaliana* | NP_200227 |
| MsCOMT | *Medicago sativa* | AAB46623 |
| SmCOMT | *Selaginella moellendorfﬁi* | GQ166949 |
| CrCOMT | *Catharanthus roseus* | AAK20170 |
| LpCOMT | *Lolium perenne* | AAD10253 |
| ZmCOMT | *Zea mays* | Q06509 |
| AtCOMT-like1 | *Arabidopsis thaliana* | NP_173534 |
| AtCOMT-like2 | *Arabidopsis thaliana* | NP_173535 |
| AtCOMT-like3 | *Arabidopsis thaliana* | NP_173536 |
| AtCOMT-like4 | *Arabidopsis thaliana* | NP_849693 |
| AtCOMT-like5 | *Arabidopsis thaliana* | NP_174579 |
| AtCOMT-like6 | *Arabidopsis thaliana* | NP_974004 |
| AtCOMT-like7 | *Arabidopsis thaliana* | NP_974076 |
| AtCOMT-like8 | *Arabidopsis thaliana* | NP_177805 |
| AtCOMT-like9 | *Arabidopsis thaliana* | NP_177876 |
| AtCOMT-like10 | *Arabidopsis thaliana* | NP_177877 |
| AtCOMT-like11 | *Arabidopsis thaliana* | NP_190882 |
| AtCOMT-like12 | *Arabidopsis thaliana* | NP_198533 |
| AtCOMT-like13 | *Arabidopsis thaliana* | NP_200192 |
| AtF5H | *Arabidopsis thaliana* | Q42600 |
| BnF5H | *Brassica napus* | AAG14961 |
| BpF5H | *Broussonetia papyrifera* | AAW50818 |
| CaF5H | *Camptotheca acuminata* | AAT39511 |
| CeF5H | *Centaurium erythraea* | AAS92625 |
| GmF5H | *Glycine max* | ABC68398 |
| ItF5H | *Isatis tinctoria* | ABL07485 |
| LeF5H | *Lycopersicon esculentum* | AAD37433 |
| LsF5H | *Liquidambar styraciﬂua* | AAD48912 |
| MsF5H | *Medicago sativa* | ABB02162 |
| NtF5H | *Nicotiana tabacum* | ABC69406 |
| OsF5H | *Oryza sativa* | NP_001065043 |
| PtF5H | *Populus trichocarpa* | CAB65335 |
| SmF5H | *Selaginella moellendorfﬁi* | EU032589 |
| ObEOMT1 | *Ocimum basillcum* | AF435008 |
| ObCvOMT1 | *Ocimum basillcum* | AF435007 |
| MdoOMT1a | Malus × domestica | KM516782 |
| MdoOMT1b | Malus × domestica | KM516783 |
| MdoOMT2 | Malus × domestica | KM516784 |
| MdoOMT6 | Malus × domestica | KM516785 |
| CbIEMT | *Clarkia breweri* | AAC01533 |
| GmMYBZ2 | *Glycine max* | ABI73970.1 |
| AmMYB308 | *Antirrhinum majus* | P81393.1 |
| EgMYB1 | *Eucalyptus gunnii* | CAE09058.1 |
| EjMYB2 | *Eriobotrya japonica* | AID56314.1 |
| CmMYB1 | *Chrysanthemum morifolium* | AEO27497.1 |
| PhMYB4 | *Petunia x hybrida* | ADX33331 |
| MusaMYB31 | *Musa AAB Group* | ANR02349.1 |
| ZmMYB31 | *Zea mays* | NP_001105949 |
| ZmMYB11 | *Zea mays* | AIB05021.1 |
| PvMYB4 | *Panicum virgatum* | AEM17348.1 |
| ZmMYB42 | *Zea mays* | NP_001106009.2 |
| ZmMYB38 | *Zea mays* | AIB04526.1 |
| AtMYB4 | *Arabidopsis thaliana* | AAS10085 |
| AtMYB3 | *Arabidopsis thaliana* | BAA21618.1 |
| AtMYB32 | *Arabidopsis thaliana* | AEE86444.1 |
| AtMYB7 | *Arabidopsis thaliana* | AEC06531.1 |
| AmMYB330 | *Antirrhinum majus* | P81395.1 A |
| AtMYB8 | *Arabidopsis thaliana* | Q9SDS8.1 |
| AtMYB6 | *Arabidopsis thaliana* | NP_192684 |
| ZmMYB8 | *Zea mays* | ADX60104.1 |
| VvMYB4 | *Vitis vinifera* | AID68565 |
| PhEOBII | *Petunia x hybrida* | ACB59077.1 |
| OsMYB4 | *Oryza sativa* | BAA23340.1 |
| EjMYB1 | *Eriobotrya japonica* | AID56313.1 |
| AtMYB123 | *Arabidopsis thaliana* | Q9FJA2.1 |
| AtMYB75 | *Arabidopsis thaliana* | Q9FE25.1 |
| AtMYB5 | *Arabidopsis thaliana* | Q38850.1 |
| VvMYB5a | *Vitis vinifera* | AAS68190 |
