# Supplementary material for: Comparative transcriptome analysis of oil palm flowers reveals an EAR-motif-containing R2R3-MYB that modulates phenylpropene biosynthesis
Source: BMC Plant Biol. 2017 Nov 23;17:219. doi: 10.1186/s12870-017-1174-4 (PMC5701422; doi:10.1186/s12870-017-1174-4)
Supplement: Supplementary file 6 — Nucleotide sequence information of phenylpropanoid pathway genes from RNA-seq. (DOCX 22 kb) [file 12870_2017_1174_MOESM6_ESM.docx]

**Additional file 6.** Nucleotide sequence information of phenylpropanoid pathway genes from RNA-seq.

>EgCvOMT1

ATGCAAGCGATGGGACTCATCCAGGAGAATCAGCCGGCCAAGGACACACTCCAAAACAAAGCCCAGCTGTGGAACCACGCCCTAAGCTTCATTAGATCCATGTCCCTCAAGTGCGCCATCGAGCTCGGCATCCCGGACGTTCTGCACAACCACGGCAAGCCCATCACTCTCTCCGAGCTTGCCACCTTCCTCTCCATCCCTCCGTCCAAAACCCCCGCCCTCGGAAGCCTCATGCGCTTGCTCGTCCACTCCGGCCTTTTCGCGAGTCACCGACACCAAGGAGGGGGAGAAGAAGAGAGTTATTTCCTCACGCCGACCTCTCAACTCCTGGTGAAGGAGGAGAAGGCGTGTATCTCGCCGTTCGCGCTGACGGCATTGAACTGGATCGTGTTGGTGCCGGGGCGCCGGCTGGGGCAGTGGTTCAGGGCCGGGGCGGCGGGGGAGACGCCGTTCGACATGGCGCACGGCAAGGGGTTATTTGGGGAGACGAACGCGAGGGCGGAGTTCAACGAGATGTTCAACGAGGCGATGGCGAGCGACGCGCGGCTGGTGACGGAGGTGGCGCTTCTGCGGCGGCAGTGGGAGGTGCTCCGCGGGGTGCGGTCGGTGGTCGACGTGGGCGGGGGCACGGGCACGGTGGCGAAATCCATCGCCAAGGCGTTGCCCGAGGTCCGGTGCATGGTGCTGGACCTGCCGCACGTGGTGGCCACGATCGACGAGGAGCAGCGGGAGGGCGTGGAGTTCATCGGTGGGGACATTTTCGAACGCATCCCTCCAGCAGATGCCGTCTTGCTCAAATGGATATTACATGATTGGAGTGATGAGGACTGTATCAAGATACTAAAGCGTTGCAAAGAAGCGATTCCTACTAAGAAAGATGGTGGAAAGGTAGTCATAATAGATGTTGTAGTTGGTGTTACGACTGATATTTCTATTTCAGTTGAATCACAACTTTTTTTGGACATGGAGATGATGATCCTTACTACGGGAAAAGAACGAAGTGAAATCGAATGGAAACGTTTATTCCATGCTGCAGATTTTAGCGACTACAAGATCACACCATCCATGGGTTTACGATCAGTCATTGAGCTGTATAACTAA

>EgCvOMT2

ATGGAATCCTTAGTTGGAGAATGTCAATCTTTAGAGCTCTTGAAAGCTCAAGCTCATTCATGGAACCACATATTCAGCTTCATAAACTCCATGTCCCTCAAGTGTGCCGTAGAGCTCGGCATAGCAGACATCATCCACACCCATGGCAAGCCAATCTCCCTCCTTGAACTAGCAACCAAACTATCCATCCCCCCCACCAGAAACAACGACTTGCGCCGCCTCATGCGCCTCCTGGTGTTCTCGGGATTCTTCGGTCGAAAGCGTGATGGGAACAGTCCCGATCACGACGACGAAGAGCTGTACATCCCCAACACCATGTCTGCCCTCCTTGTCAAGGAGAAGGATGCGAACGTCTCGTCGTTCCTTCTGATGCTGGACCAAATGTTGCTCAACCCATGGTATAACCTCAGTAGTTGGTTCAAGCAAGATGAGCTGCCCACGCCCTTCGAGGTAGCACATGGAAAGGGTGTTGGGGAGGCCACCAGCCAAACCCCGGGCTTCGATGGCTTGGTGAACGAGGGGTTGGCAAGCGACTCTCAGTTCGTGGCCAAACTGGTCGTCGAGGAGTGCGGCGAGGTCTTCAGAGGCCTGAGGTCGCTGGTGGACGTTGGTGGGGGGACGGGCACGATGGCCCGGGCCATCGCGGANGCCTATCCACAGGTGAAGTGCACCGTCCTTGACCTGCCCCACGTGGTAGCGAGCTCGGAAGCGAGCGAGGCGGTCCAGCTTGTCGCGGGAGATATGTTTCAGTATATCCCTCCTGCTGATGCAGTGCTGCTCAAGTGGGTGCTACATGATTGGAGCGACGAGGACTGCGTAAACATACTGAAACAATGCAAGAAGGCTGTTCCATCCAAAGAAGAGGGGGGAAAGGTGATCATTATTGAAATGGTAGTTGACTTGGATATTGGCTTTCCTGAGTTGGTTGAAACACAGCTACTCTTTGACATGCACATGATAGTCCACACCACAGGGAAACAAAGAAAGGAATGCGAATGGAAAAAAAATTTTACTGATGCAGGTTTTACCGACTACAAAATCATACCGGCATTAGGCGTGCGTTCAGTTATCGTGGCTTATTATTAA

>EgEGS

ATGGAAGCAGCAAAGAAGGGCAAGATCCTAATCATCGGAGCGACGGGGTACCTTGGAAAGTACCTCACTGAGGGGAGCTTGAGATTAGGTCACCCTACCTTCATTCTAGTGAGGGAAAGCACCATTAACACTAATTCAGAAAAGGCCAAGGTCATCAAAGATTTCAAAGATGCTGGAGCCACTGTGCTCTATGGAGATTTGCATGACCACGAGAGCTTGGTGAAGGCAATTAAGCTGGTGGATATCGTCTTCTCGACGATGGGCCATGAGCACCCAGAGCAGCTGGCGAGTCAGATTAACATCGTCTCAGCCATTAAAGAGGCTGGAAATGTGAAGAGATACTTTCCATCAGAGTTTGGGGTTGATGTCGGTCGAATCCAACTTTTGGAGCCAGCAAAAAGCGTGCTCGGAATAAAGGTTAAAATCAGACAAGCAATAAGGATGGCAGGAATTCCCTTCACCTTTGTCTCAAGCAATCTTTGTGCAACATACTTCCTTTCTAGACTTGGGCAAGTTGAAGGTGATGGGACCCCAAAAGATGAAGTCTCCATCATGGGGGATGGAAACGCCAAAGTTATTTTTGTGTGCGAGCAAGATATGGCAACATATGCCATCAAAGCTGCAGAAGACCCAAGAACTCTGAACAAGATCCTATATGTGAGACCACCAGCTAACATCTACTCTCAAAATGAGCTTGTATCCCTTTGGGAGAAGAAAACTAAGAACACCCTTAGAAGGACTTATCTTTCTGAAGAAGAGGTTATGAAGAAGATCCAAGATGCTCCGTATGCGGTCTCTTTTATTTATGCTATCGCACACGCATGCTTCATGAAAGGAGAGTTGACCAACTTTGAGATAGATCCCTCAATGGGAATAGAGGCCTCAAATCTCTACTGCGAAGTCAAGTATATCACCGTCGATGAATATCTTGACAAATTCATATGA

>EgCOMT

ATGCTGGCCTTTGCAGACTCCATGGTCCTCCGCTGCGCCGTGGAGCTCGGCATCGCCGACATCATCCACCAGCACGGAGAGCCCCTCTCCCTCCCCCACCTCGCCTCCCTCATCCCCTCCCCGCCCCCCAACCCCACTTCCTTCCTCCGCATCATGCGCTTCCTCACCCACAAGAAGATCTTCAGCACCCATTCCCACCCCGACACCGGCGAGACCCTCTACGGCCTCACCCCGGCCTCCCGGGTCCTCGTCCGCTCCTCCGACTTCTCTCTCGTCCCCATGGTCCTCTTCGAGACCCACCCCTTCCCCATCGCGGCATGGCACAGCTTCAGCGCCAGCGTCCGCCAGGGTGGTGCGCCGTACATCATGGCGCACGGCATGGACATGCTGTTCGGCACCGCGGCGACGAACCCCGAGTTCAACGCGGTGTTCAACAACGCGATGGCGTGCACCGCCAAGGGCACCCTCGACGCTGTCATCTCGGCGTACGGGGATGGTTTCGCGGGCTTGGGCTCGCTTGCGGACGTCGGCGGGGGGACCGGGGCGACGCTGGGGGAGATTGTGAAGTTGCATCCTGGCATCAAGGGGATCAACTTCGATCTGCCGCATGTCATCGAGGGTGCGCCGGAGTACCCCGGTGTGACTCATGTTGCCGGGGATATGTTTCAGACCATCCCTCCAGCGGATGCTGTGTTCATGAAGACGGTGATGCATTGCTTTGGAGATGAGGAGTGTGAGAAGGTCCTGAGGAACTGCCGAGGTGTTATACCAGAGAAGACCGGGAAGGTTATAATTGTGGACGTGGTATTGTACCCGGATGACAATAGCCCATTGGGAGACATACGGGCGAAGTTTGACATGCTGATGTTTGCCTACACATCAACTGGAAAGGAGAGGACCGAAGAGGAGTGGAAGAAGCTTCTGAAAGCGAGTGGCTTTCCTCGGTGGAACATTATTAGAATTCCTGCCATATGGTCCATCATTGAAGCATTCCCTGAGTAA

>EgF5H

ATGGATTGGCTCCAAGAGTCTACTATCATGAGCTTCATATTATTCTTCTTGATACCTCTAGCTCTCTTGTTTGTTCTCTCCACGAGGCGCCGGCGGGAGCTCCTGTTTCCGCCGGGGCCCCGTCAACTGCCGATCATCGGAAACATGCTGATGATGGACCAGCTGACCCACCGCGGCCTCGCCAAGCTCGCAGACAAGTACGGTGGCCTGTGCCACCTCCGCCTCGGCTACCTCCATGCCTTCGCGGTGTCGACGCCGGAGATGGCCCGAGAAGTCCTCCAGGTTCAGGACAACGTCTTCTCCAACCGACCGGCCACCATGGCCATCACCTATCTCACCTACGACCGCTCCGATATGGCCTTCGCCCACTACGGTCCCTTCTGGCGCCAGATGCGCAAGCTCTGTGTCATGAAGCTCTTCAGCCGCAAGTGCGCCATGTCGTGGGCCGCCGTCCGCGAAGAGATCGAAGTCATGATCCATGCCGTCGCTGGCCGGAAAGGCACCACCGTCAACCTCGGCGAGCTCATCTTCAACCTCACCATGAACATCACCTTCCGAGCAGCTTTCGGAACCCCGAACCACGAGAACCAGGATGAGTTCGTCTCAATACTACAAGAGTTCTCCAAGCTCTTCGGCGCCTTCAACATCGGGGACTTCATCCCGTGGCTCAGCTGGATGGACCCCCAAGGCATCAACAAGAGGCTCAACGTTGCGCGGGCCGCCCTCGACCGCTTCATCGACAAGATCATCGACGAGCACATGGCGAACCCGAAGGCGACCGATGCGGAGGACGCCGACATGGTCGACGACATGCTCGCGTTCCTAGATGATCATTCTCGTGTTAGCAAGCAAGGTGGTGAGAAGGATGAGCTCCAGGGAAACCTCAAGCTCACCAGGGATAACATCAAGGCTATAATCATGGATGTGATGTTCGGTGGCACGGAGACTGTGGCCTCGGCGATAGAATGGACCATGGCAGAGCTAATGAGGAGCCCCGACGACCTAAAGCGGGTGCAGCGAGAGCTGGCTGACATAATTGGCCTGGACCGCAAAGTCCACGAGAACGACCTCGACAGGCTCCTGTTCCTCAAGTGTGCCACCAAAGAGACTCTCCGCCTCCACCCTCCTATCCCACTCCTCCTTCATGAGACCGCCGTGGACTGCAAGGTCGACGGCTACTTTATCCCTAAGAAATCAAGAGTCATGATCAACGCCTGGGCCATCGGCCGCGACAAATCGGCATGGAGGGATGCTGACAAGTTCCGGCCATCGAGGTTCGCCTCGGGCGGCGACAGCACGGGCATCGACTTCAAGGGGAACTTCTTCGAGCTCATACCGTTCGGGTCGGGGCGGCGGTCATGCCCGGGGATGCAGCTGGGACTGTACGCACTGGAGCTGGCGGTGGCGCAGCTCCTGCACTGCTTCAATTGGGCCCTGCCGGATGACATGAAGCCGAGCGAGCTCGACATGGGGGATGTCTTTGGGCTCACCGCGCCGAGGGCGGTCCGGCTCACTGCAGTGCCCACTCCACGACTCTCTTGTGCACTATACTAA

>ObCOMT1

ATGGGCTCAGCAACCAACACTCCCCAAATTAATTCCGATGAGGAAGAAAATTTCCTCTTCGCCATGCAGCTGGCAAGCGCTTCCGTACTGCCAATGGTGCTTAAATCCGCCATTGAGCTCGACTTGCTCGAGCTCATCAAGAAATCCGGCGCCGGCGCCTTTGTTTCGCCGGTCGACCTCGCCGCGCAGCTCCCCACCACCAACCCCAACGCGCACGTCATGCTCGACAGAATTCTCCGCCTTCTCACCAGCTACGCCATCCTCGAATGCCGCCTGAAAACGCTCCCCGACGGCGGAGTGGAGCGCCTCTACGGGTTGGCGCCGGTGTGCAAGTTTTTGACTAAGAACGAGGATGGAGTTTCCATGGCTCCTTTGACGCTCATGAATCAAGATAAAGTGCTCATGGAGAGCTGGTACCATCTAAAAGATGCAGTTCTTGATGGAGGGATTCCCTTTAACAAAGCCTATGGAATGACTGCATTTGAGTATCATGGGACTGATCCAAGATTTAACAAAGTGTTTAACCAAGGAATGTCTAATCACTCAACCATTACTATGAAGAAGATTCTAGAAACATACACCGGTTTTGATGGCCTCAAAACTGTGGTTGATGTTGGAGGAGGAACAGGGGCCACACTCAACATGATTGTCTCCAAGTATCCATCTATTAAGGGCATCAATTTTGATCTCCCTCATGTCATTGAAGATGCACCATCTTATCCAGGAGTGGAGCATGTTGGTGGGGACATGTTTGTGAGTGTGCCTAAAGGAGATGCTATCTTTATGAAGTGGATTTGCCACGATTGGAGTGATGAGCACTGCGTGAAATTCTTGAAGAATTGCTATGATGCACTCCCACAAAATGGGAAAGTGATATTGGCTGAGTGTGTTTTACCTGAGGCGCCAGACACAGGGCTGGCCACCAAGAATGTAGTCCACATTGATGTCATCATGTTGGCTCACAACCCTGGCGGTAAGGAAAGGACAGAAAAGGAATTTCAAGGCTTGGCCAAAGCTGCTGGCTTCAAACAATTCAACAAAGCTTGCTGTGCTTACAATACTTGGATCATGGAGCTTCTCAAATGA

>ObCOMT2

ATGGATGAGAAGGGAGAAGACGAAGGCGCTTTCCTAATGGCTATGCGACTGGCTGTGGGTTCAGTGCTTCCAATGGCTCTTCGATCCGCCATAGAACTGGATCTTCTCCAACTCATCAAGAAATGTGGGTCGGAAGGAGCTTCTGCTTCTCAGCTTGCTGCACTGCTTCCCACAACCAACCCCGATGCCCCCGACATGATCGACAGAATTCTCCGACTCCTGGCCGCCCACTCTATCCTCATTTGTGAGGAGCGGCGCTACACCCTGGCGCCGGTGTGTAAGTATCTGACTAGGGATGAGAATGGGGTTTCTCTAGCGCCTGTTTCGCTCTTGATCCAAGGCAAAGTCGTGATGGAGTCATGGTACCAGACGAGTGATGCGATTCTTGGAGGAGGAGTCCCCTTCGACAGAGCGCATGGCATGAATGCATTTGAATACCCGGTGAAGGACCCTGAATACAACACGGTTTTCAATGAAGCTATGCATCAACAGTCCACCATTTTTATGAAGAGAATTCTTGAGATTTACAATGGATTTGAGGGCCTGAAATCCTTGGTGGATGTTGGAGGTGGAACAGGGGCTTCCTCCAAGCTCATCATATCCAGATTTCCATCCATCAAGGCCATCAATTTTGATTTGCCCCATGTTATTCAAAATGCTTCACCCCATCCCGGAGTGGAGCACGTTGGTGGCGACATGTTCGTTCAAGTGCCCAAAGCTGATGCCATTTTTATGAAGTGGATTTGCCATGACTGGAGCGATGCACATTGTGCCAAACTATTGAGAAATTGCTATGAAGCCCTTCCAGAAAACGGGAAACTCATAATTGCCGAAAGTATCCTTCCGGAGGATCCAAACAGCGAGGAGGCTGCCATGGGTTGCATCGGCGATGTGATCATGCTCACCGTTAATCCGGGAGGGAGAGAAAGGACACAAAAGGAATTTCAGGCGTTGGCTTTGCAAGCAGGCTTTAAACAACTCATCAAAGTTTGTGTCGCTTTTAATATTTGGATCATCGAACTTCATAAATCATATTAA

>ObF5H

ATGAAAATGGTTAATCTCCTGGAAACCAACAACCCCTTGCTCTTCATCTCCTTCACAATCCCTCTATTTATCTTCTTCTTCTTCCTATCAAGATTCCGACGTAAACGGTATCCTCCGGGGCCACGTGGATGGCCCGTAATAGGCAACATGGGCCTGATGGACCAACTGACCCACCGTGGGCTAGCCAAGCTGGCCAAGAAATACGGCGGAATCTTCCACCTCCGCATGGGGTTCGTCCACATGGTGGCGGTGTCCAGCCCCGACGTGGCGCGCCAAGTCCTCCAAGTCCACGACAACATCTTCTCCAACCGCCCCGCTACCATCGCCATCAGTTACTTAACCTACGATAGGGCTGACATGGCCTTCGCCCACTACGGGCCCTTTTGGCGCCAAATGCGCAAGCTCTGTGTGATGAAGCTCTTCAGCCGTAAACGGGCCGAGTCCTGGGACTCCGTCCGTGATGAGGTCGACGACATGGTGCGGGCCGTGGCTACCAGTAGCGGCACGGTCGTCAATATCGGCGAGCTAGTTTTCGGGCTCACCAAGAATATAATATACCGGGCTGCCTTCGGGTCGAGTTCCCATGAGGGACAAGATGATTTCATTAAGATTTTGCAGGAATTCTCCAAGTTGTTTGGAGCCTTTAATATCGCGGATTTCATTCCATGGTTGGGGTGGATTGATCCGCAGGGATTGAACGGCAGGTTGATCAAAGCGCGGGCCGAGCTCGATGGATTCATCGACACCATAATTGATGATCATATGCAGAAGAGGCGGCCTGAAAATGGCGGCGGTGAGGCGGTTGAGTCTGATATGGTGGATGAGTTGCTTGTTTTTTACAGTGAGGAGGCGAAGCTTAATTTAGAGTCTGAGGATTTGCAGAATTCCATCAAGCTTACCAGGGACAACATTAAGGCCATTATTATGGATGTAATGTTCGGTGGGACGGAGACGGTGGCGTCGGCAATCGAGTGGGCCATGACGGAGTTAATGAGAAGCCCAGAAGATCTGAAGCGGGTCCAACAAGAGATGATGGATGTTGTTGGCCCGACCCGGAAAGTCGAAGAGTCCGATTTCGAGAAACTCACCTACCTCCGCTGCTGCCTCAAGGAAGTCCTCCGCCTCCACCCGCCCATCCCCCTCCTCCTCCACGAGACCTCCGACGACGCCGTCATCTCCGGCTACCACGTTCCCGCCAAGTCGCGCGTCATGATCAACGCCTGGGCCATCGGCCGCGACCCGAGCGCGTGGGAGGACGCAGAGTCCTTCAAGCCCTCGCGATTTCTGAGAGACGGCGTCCCCGATTTCAAGGGCGGCAACTTCGAGTTCATCCCGTTCGGGTCGGGGCGGCGGTCCTGCCCCGGCATGCAGCTGGGGCTGTACGCGCTGGAGGTGGCGGTGGCGCATCTCCTCCATTGTTTCACGTGGGAATTGCCGGATGGAATGAAGCCCAGCGAGATGGATATGGATGATGTGTTTGGCCTCACGGCGCCACGCGCCACCAGGCTCATGGCCGTGCCCACGCCGCGGCTGCTCTGCCCCCTCTACTAG
