# Supplementary material for: Comparative transcriptome analysis of oil palm flowers reveals an EAR-motif-containing R2R3-MYB that modulates phenylpropene biosynthesis
Source: BMC Plant Biol. 2017 Nov 23;17:219. doi: 10.1186/s12870-017-1174-4 (PMC5701422; doi:10.1186/s12870-017-1174-4)
Supplement: Supplementary file 7 — GC-MS analysis of phenylpropenes in sweet basil leaves. (DOCX 74 kb) [file 12870_2017_1174_MOESM7_ESM.docx]

**
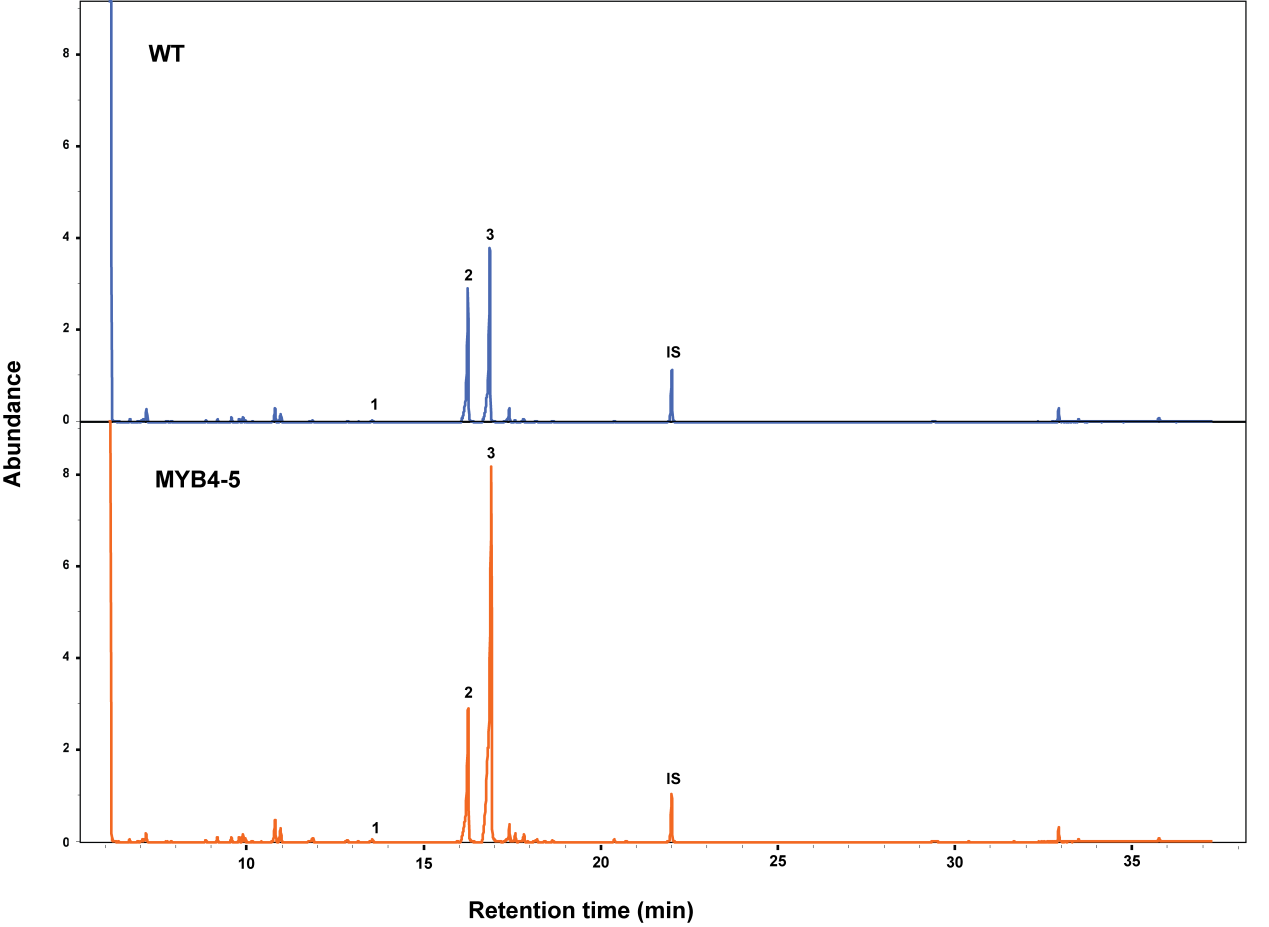
**

**Additional file 7.** GC-MS analysis of phenylpropenes in sweet basil leaves.

1, methylchavicol; 2; Eugenol; 3, methyleugenol; IS, internal standard; WT, wild type; MYB4-5, EgMYB4 over-expressing sweet basil line.
