# Supplementary material for: Comparative transcriptome analysis of oil palm flowers reveals an EAR-motif-containing R2R3-MYB that modulates phenylpropene biosynthesis
Source: BMC Plant Biol. 2017 Nov 23;17:219. doi: 10.1186/s12870-017-1174-4 (PMC5701422; doi:10.1186/s12870-017-1174-4)
Supplement: Supplementary file 8 — Expression levels of predicted flavonoid biosynthesis genes in oil palm leaves and flowers. (DOCX 20 kb) [file 12870_2017_1174_MOESM8_ESM.docx]

**Additional file 8.** Expression levels of predicted flavonoid biosynthesis genes in oil palm leaves and flowers.

| transcripts | leaf | female flower | male flower | annotation |
| --- | --- | --- | --- | --- |
| comp69356_c0 | 0 | 2.34 | 6.46 | flavonol synthase/flavanone 3-hydroxylase, putative, expressed |
| comp17707_c0 | 0 | 1.72 | 4.83 | flavonol synthase/flavanone 3-hydroxylase, putative, expressed |
| comp49906_c0 | 3.29 | 4.75 | 7.18 | naringenin,2-oxoglutarate 3-dioxygenase, putative, expressed |
| comp36591_c0 | 0 | 3.58 | 2.87 | flavonol synthase/flavanone 3-hydroxylase, putative, expressed |
| comp45931_c0 | 0.98 | 2.33 | 2.62 | chalcone synthase, putative, expressed |
| comp89241_c0 | 1.49 | 0 | 0.20 | dihydroflavonol-4-reductase, putative, expressed |
| comp42140_c0 | 3.70 | 2.34 | 2.10 | dihydroflavonol-4-reductase, putative, expressed |
| comp24013_c0 | 5.34 | 0 | 3.21 | naringenin,2-oxoglutarate 3-dioxygenase, putative, expressed |
| comp39145_c0 | 2.33 | 0 | 0 | chalcone isomerase 3, putative, expressed |
| comp42653_c0 | 2.64 | 0 | 0.11 | dihydroflavonol-4-reductase, putative, expressed |
| comp42868_c1 | 6.63 | 0 | 3.59 | flavonol synthase/flavanone 3-hydroxylase, putative, expressed |
| comp33521_c0 | 3.18 | 0 | 0 | dihydroflavonol-4-reductase, putative, expressed |
