# Supplementary material for: Comparative transcriptome analysis of oil palm flowers reveals an EAR-motif-containing R2R3-MYB that modulates phenylpropene biosynthesis
Source: BMC Plant Biol. 2017 Nov 23;17:219. doi: 10.1186/s12870-017-1174-4 (PMC5701422; doi:10.1186/s12870-017-1174-4)
Supplement: Supplementary file 9 — DNA primers used in this study. (DOCX 21 kb) [file 12870_2017_1174_MOESM9_ESM.docx]

**Additional file 9.** DNA primers used in this study.

| **Gene** | **Sequence (5’-3’)** | **Purpose** |
| --- | --- | --- |
| EgCvOMT1-F | GTTATTTCCTCACGCCGACC | QRT-PCR |
| EgCvOMT1-R | CGTCTCCCCAAATAACCCCT | QRT-PCR |
| EgCvOMT2-F | CAAGGAGAAGGATGCGAACG | QRT-PCR |
| EgCvOMT2-R | CACGAACTGAGAGTCGCTTG | QRT-PCR |
| EgCVS-F | TTAGGTCACCCTACCTTCATTCT | QRT-PCR |
| EgCVS-R | CTTCACATTTCCAGCCTCTTTA | QRT-PCR |
| EgMYB-F | CGGACTTCACCATCACCTTT | QRT-PCR |
| EgMYB-R | CCAAGCTGCAGTTGAAACAA | QRT-PCR |
| EgMYB-YFP-F | AGTCTCCTAGGAATGGGAAGGTCTCCATGC | Localization |
| EgMYB-YFP-R | AGTCGCCCGGGATTCCATTTCAACTGTTCT | Localization |
| PET-EgMYB-F | GTACTCATATG ATGGGAAGGTCTCCATGC | Protein expression |
| PET-EgMYB-R | GTACAGCGGCCGC TTCCATTTCAACTGTTCT | Protein expression |
| EgF5H-F | TCGTGTTAGCAAGCAAGGTG | QRT-PCR |
| EgF5H-R | AGGCCAATTATGTCAGCCAG | QRT-PCR |
| EgCOMT-F | GTGAAGTTGCATCCTGGCATCAAG | QRT-PCR |
| EgCOMT-R | TCTGGTATAACACCTCGGCAGTTCCT | QRT-PCR |
| AC-Ⅳ | GAGGCCCATAAACCAAACGTAGAAAAG | EMSA |
| AC-Ⅴ | GTTATCCGTTCGCAACAACCCGCCATATCAACCAAG | EMSA |
| EgF5H Promoter-F | GAGGCCTGCAGTTATCAGCACGTACTTGTTG | GUS assay |
| EgF5H Promoter-R | GCGGCGTCGACTTTCCCTAAAAGGTATTTCT | GUS assay |
| EgCOMT Promoter-F | CACCGGAGAATGATTGGACCTAAACT | GUS assay |
| EgCOMT Promoter-R | GCTTTCTATCCCTCGCACTT | GUS assay |
| pK7WG2D-EgMYB1-F | CACCATGGGAAGGTCTCCATGCTGCGA | Expression in basil |
| pK7WG2D-EgMYB1-R | TCATTCCATTTCAACTGTTCTAT | Expression in basil |
| ObF5H-F | GATTTCATTCCATGGTTGGG | QRT-PCR |
| ObF5H-R | CCCTGGTAAGCTTGATGGAA | QRT-PCR |
| ObCOMT1-F | GATGGCCTCAAAACTGTGGT | QRT-PCR |
| ObCOMT1-R | AATCGTGGCAAATCCACTTC | QRT-PCR |
| ObCOMT2-F | TGCATCAACAGTCCACCATT | QRT-PCR |
| ObCOMT2-R | AAAAATGGCATCAGCTTTGG | QRT-PCR |
| EgUbiquitin-F | CAGTAGCTGATGGGTTGGGT | QRT-PCR |
| EgUbiquitin-R | CACAGACAGGGCTATGAGCA | QRT-PCR |
| ObEF-F | AATGGCAAAAAGCTCGAAGA | QRT-PCR |
| ObEF-R | TCGCAGACATGACAGACACA | QRT-PCR |
